# Using a female-specific isoform of *doublesex* to explore male-specific hearing in mosquitoes

**DOI:** 10.1016/j.isci.2025.113330

**Published:** 2025-08-28

**Authors:** Matthew P. Su, Marcos Georgiades, Marta Andrés, Jason Somers, Judit Bagi, YuMin M. Loh, Yifeng Y.J. Xu, Kyros Kyrou, Andrea Crisanti, Joerg T. Albert

**Affiliations:** 1 Ear Institute, University College London, 332 Gray’s Inn Road, London WC1X 8EE, UK; 2 The Francis Crick Institute, 1 Midland Road, London NW1 1AT, UK; 3 Institute for Advanced Research, Nagoya University, Nagoya 464-8601, Japan; 4 Graduate School of Science, Nagoya University, Nagoya 464-8601, Japan; 5 Institute of Transformative Bio-Molecules (WPI-ITbM), Nagoya University, Nagoya 464-8601, Japan; 6 Animal Health Research Centre, National Institute for Agricultural and Food Research and Technology, Spanish National Research Council (CISA-INIA-CSIC), 28130 Valdeolmos, Spain; 7 Department of Life Sciences, Imperial College London, London, UK; 8 Department of Molecular Medicine, University of Padova, Padua, Italy; 9 Cluster of Excellence Hearing4all, Sensory Physiology & Behaviour Group, Department for Neuroscience, School of Medicine and Health Sciences, Carl Von Ossietzky University Oldenburg, Carl Von Ossietzky Str. 9-11, 26111 Oldenburg, Germany

**Keywords:** Developmental anatomy, Ecology, Molecular Genetics, Sensory neuroscience, Evolutionary Developmental Biology

## Abstract

Animal reproduction relies on elaborate divisions of labor, and multiple dimorphisms, between the sexes. Primary dimorphisms affect core reproductive elements; secondary dimorphisms affect indirect traits, including complex behaviors. Male disease-transmitting mosquitoes locate female mating partners acoustically before copulation (phonotaxis). Male ears—and hearing performance—have thus evolved to become substantially more complex, partly due to sex-specific expression of the gene *doublesex* (*dsx*). *dsx* links primary and secondary dimorphisms: spermatogenesis and ear morphogenesis share considerable molecular overlap; both depend on *dsx* expression patterns. Here, we combine functional-anatomical analyses of mosquito ears with transcriptomic profiling to dissect *dsx*-dependent hearing in the malaria mosquito *Anopheles gambiae*. Female *dsx* mutant ears are anatomically and functionally intersex, although they still completely lack some male-specific characteristics. By cross-linking our auditory findings to the genetic bases of spermatogenesis we advance the molecular understanding of sex-specific hearing mechanisms in insects, highlighting the special roles of ciliary factors.

## Highlights

• Deletion of sex-specific *doublesex* isoform alters hearing in female *Anopheles gambiae*
• Mutant female ears are partially masculinized but lack self-sustained oscillations (SSOs)
• Transcriptomic analyses link “auditory maleness” to ciliary function
• Comparing transcriptomes of ears and testes finds overlap in ciliary motility machineries

## Graphical abstract

**Figure:**
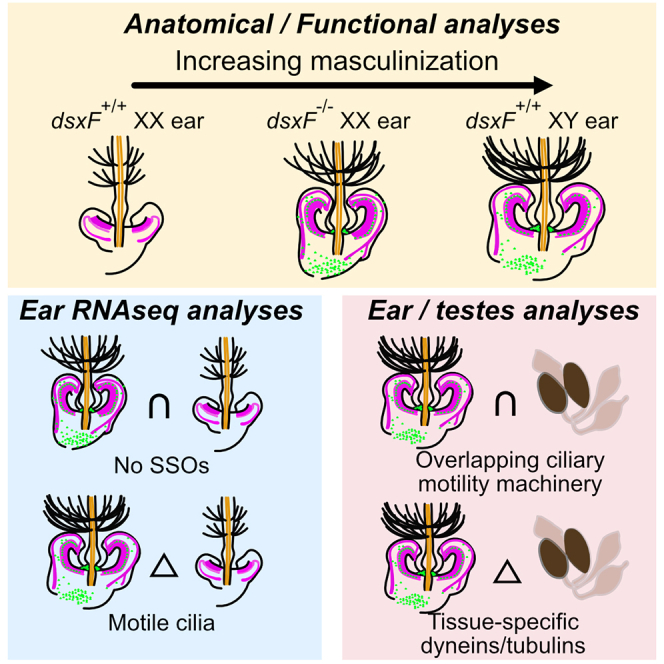

## Introduction

In insects, sexually dimorphic behaviors are supported by sex-specific anatomical structures. An exemplary demonstration is the highly sexually dimorphic hearing system of male mosquitoes, whose function underlies mating. Anatomically, the male mosquito ear is a complex structure, containing many thousands of neurons and multiple populations of auditory efferent fibers from the central nervous system.^1^^,^^2^ This male-specific complexity is required for successful copulation, as male mosquitoes detect and locate conspecific females within the crowded environment of (male-dominated) swarms.^3^ Male phonotactic attraction to female flight sounds is augmented via spectral matching between female wing beat frequencies (WBFs) and natural vibration frequencies of the male flagellar ear.^4^ Phonotaxis appears highly conserved across multiple mosquito species,^5^ although attempts at applying this knowledge for use in vector control have yet to be maximally exploited.^6^^,^^7^

In contrast to males, females from disease-transmitting mosquito species do not appear to demonstrate positive phonotaxis.^8^ Immunological and neuroanatomical assays have enabled the description of female auditory features, reporting an ∼50% reduction in the number of neurons and a more limited (or close to non-existent in some species) efferent network when compared with males.^1^^,^^9^ Despite this numerical reduction relative to males, the female mosquito ear still stands among the most complex insect hearing organs. Yet, female hearing-related behaviors have still to be elucidated, although numerous theories have been devised ranging from host seeking to mate selection.^5^^,^^8^^,^^10^ Furthermore, both sexes may utilize hearing for predator avoidance, indicating potential conservation of auditory processing,^11^ although it should be noted that selection pressures related to predator avoidance may be greater for females due to their broader activity patterns and resulting greater exposure.^12^ The precise molecular substrates responsible for the anatomical and behavioral differences between male and female ears remain unknown.

Beyond differences in auditory neuroanatomy and behavior, there are clear dimorphisms in hearing function between male and female ears.^9^ Male ears are tuned to higher frequency sounds than females at both mechanical and electrical levels.^10^ Males also show a unique hearing state, referred to as self-sustained oscillations (SSOs), in which the male flagellum—in the absence of any external stimulus—becomes an almost mono-frequent active oscillator with displacements orders of magnitude larger than in non-SSO states.^9^^,^^13^ SSOs have been hypothesized to play a crucial role in mosquito courtship by entraining to, and enhancing, the flight tone of the female in the male ear.^14^ Onset/offset of SSOs appears controlled by the auditory efferent system and is powered by Johnston organ (JO) neurons in a metabolic energy-dependent process.^9^^,^^13^ SSOs are restricted to males, with males from some species appearing to more readily demonstrate SSOs than others, although the anatomical and genetic factors that form their basis are unknown.^9^

**Figure 1:**
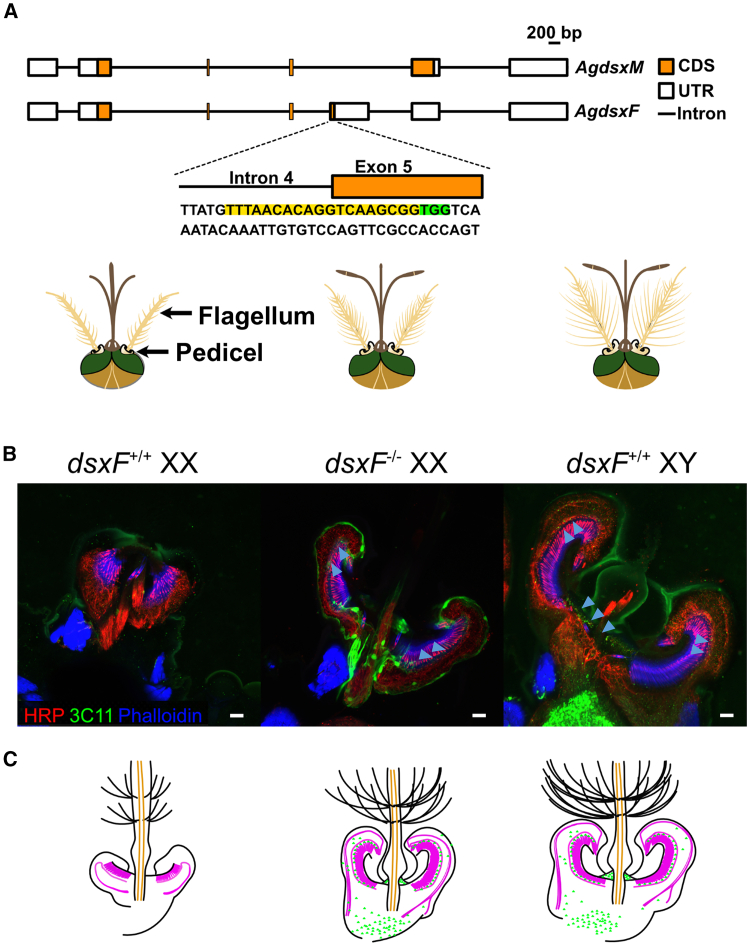
Altered auditory efferent innervation and JO size in *dsxF*^−/−^ female mutants (A) (*Above*) Schematic of male (M) and female (F) *Agdsx* isoforms (*AgdsxM* and *AgdsxF*, respectively); (*Below*) Schematic of *dsxF*^+/+^*XX* (left), *dsxF*^−/−^*XX* (center), and *dsxF*^+/+^*XY* (right) head anatomy based on previous publications. (B) Horizontal sections of *dsxF*^+/+^*XX* (left), *dsxF*^−/−^*XX* (center), and *dsxF*^+/+^*XY* (right) JOs stained with presynaptic marker 3C11 (anti-synapsin, green) to label presynaptic efferent terminals within the JO and counterstained with neuronal marker anti-HRP (red) and phalloidin (blue) to label actin-based rods in scolopale and cap cells that surround auditory cilia. 3C11 also stains muscle motoneuron innervation in scape. Scale bar, 10 μm. Arrows highlight pre-synaptic efferent terminals picked up by 3C11 antibody. See also Figure S1A for split channel images and Figure S1B for further examples of sections. (C) Schematic representations of each genotype’s JO anatomy based on (B) as well as prior images of flagellar anatomy provided in Kyrou et al.^15^

Sex-specific alternative splicing of *doublesex* (*dsx*), a key molecular switch in the insect sex determination cascade, regulates somatic sexual differentiation (Figure 1A), giving rise to sexually dimorphic morphological features.^15^^,^^16^^,^^17^ *dsx* plays an important role in sexual reproduction and in particular, the development of insect gonads.^15^^,^^18^^,^^19^ Intriguingly, male sperm and mosquito auditory neurons are the only two specialized cell types that contain motile cilia, providing an interesting connection between two defining aspects of male mosquitoes (phonotaxis and sperm motility).^14^^,^^20^

Previous work in *Anopheles gambiae* (*An. gambiae*) found that a loss-of-function mutation in the female-specific exon of *dsx* (hereafter referred to as *dsxF*) produces females with intersex morphological characteristics, including masculinization of the external ear morphology and internal/external reproductive structures.^15^ The shared motile cilia machinery between sperm and auditory neurons raises the question of how sex-specific isoforms of *dsx* differentially regulate the development of these tissues to support sex-specific reproductive and hearing behaviors. Although male ejaculate has been extensively researched to identify factors involved in regulating female behavioral changes post-copulation,^20^^,^^21^ the molecular factors of male hearing have remained largely unexplored. Identifying common genes shared between the two tissues could help find new effective vector control target genes that ideally simultaneously inhibit male hearing and reproduction.

This prior generation of an *An. gambiae dsxF* knockout mutant line offers an ideal tool for investigations of sexual dimorphisms in mosquito auditory systems.^15^ In addition to the aforementioned intersex phenotypic differences reported for *dsxF*^−/−^ *XX* mutants,^15^ the mutation produces an apparent dose-dependent masculinizing effect on female WBFs.^22^ Importantly, this *dsxF* mutant line has also been suggested for release as part of a gene drive–based mosquito control program.^15^^,^^23^ Given the importance of hearing for mosquito mating (and thus reproduction^24^), it is vital to test auditory function in these mutants before release, particularly in light of the aforementioned changes in WBF.

Here, we investigated the auditory anatomy and function in *dsxF*^−/−^ mutants to better understand the molecular mechanisms underlying sexual dimorphisms in mosquito hearing. We found significant differences in ear anatomy in *dsxF*^−/−^ *XX* mosquitoes when compared with both male and female controls, with mutants exhibiting an intersex phenotype in terms of efferent terminals in the JO, as well as pedicel size. Functional experiments found altered and intersex mechanical and electrical tuning frequencies in *dsxF*^−/−^ *XX* mutants, reflecting incomplete masculinization. By performing RNA sequencing (RNA-seq) analyses of *dsxF*^+/+^ *XY*, *dsxF*^+/+^ *XX,* and *dsxF*^−/−^ *XX* ears, we identified numerous promising gene candidates upregulated in *dsxF*^+/+^ *XY* and *dsxF*^−/−^ *XX* compared with *dsxF*^+/+^ XX mosquitoes, which may underlie the auditory masculinization. Notably, through intersectional analyses of differentially expressed genes, we identified factors that may influence SSOs.

Finally, our bioinformatic analyses showed that genes and Gene Ontology (GO) terms related to motile cilia machinery (microtubules, dyneins, etc.) appeared enriched in *dsxF*^+/+^ *XY* and *dsxF*^−/−^ *XX* ears when compared with *dsxF*^+/+^ *XX*. Given the importance of ciliary factors for sperm motility and the role of *dsx* in modulating both ear morphogenesis and spermatogenesis, we compared the auditory transcriptome with published testes data. Our analyses identified both tissue-specific ciliary factors and factors shared between the two tissues, suggesting that the basal molecular machinery for ciliary motility might be conserved between sperm and auditory neurons with tissue-specific adaptations being conferred by sets of specialized factors.

## Results

### *dsxF*^−/−^*XX* mutant ears are significantly different in size and structure to both *dsxF*^*+/+*^*XY* and *dsxF*^*+/+*^*XX* ears

The mosquito ear is composed of a feathery flagellum attached at the base to the JO, the site of auditory mechanotransduction, which is housed in the pedicel (Figure 1B).^1^^,^^25^^,^^26^ The JO contains not only thousands of neurons but also sexually dimorphic efferent terminals originating from the brain.^1^ Anatomical differences between the sexes underlie differences in function and thus behavior. Previous reports have highlighted differences in external ear structure in *An. gambiae dsxF*^−/−^ *XX* mutants, including an increased pedicel size and density of fibrillar hairs,^15^^,^^22^ but have not investigated potential changes in neuronal architecture.

To investigate changes in ear anatomy resulting from the *dsxF* mutation, we used immunohistochemistry to investigate JO neuroanatomy.^2^ *dsxF*^+/+^ *XY* showed extensive efferent innervation in their JOs, whereas no such innervation was observable in *dsxF*^+/+^ *XX* JOs (Figure 1B; individual channels shown in Figure S1A). On the other hand, the anatomy of *dsxF*^−/−^ *XX* JOs was clearly distinct from those of female controls (Figure 1B; further examples included in Figure S1B). *dsxF*^−/−^ *XX* JOs contained not only many more neurons than in *dsxF*^+/+^ *XX* JOs but also efferent terminals. Still, *dsxF*^−/−^ *XX* JOs remained distinct from control male JOs, with the number of neurons and the magnitude of the efferent populations reduced in *dsxF*^−/−^ *XX* mosquitoes (Figures 1B and 1C).

In addition to differences in JO anatomy and the density of fibrillar hairs, there was a significant difference between the female genotypes in terms of flagellar length (Figure S2A; Mann-Whitney test with Bonferroni correction; *p* = 1.458 × 10^−5^). *dsxF*^+/+^ *XY* flagella were longer than both *dsxF*^+/+^ *XX* and *dsxF*^−/−^ *XX* flagella (Mann-Whitney tests with Bonferroni corrections; *p* < 2.2 × 10^−16^ for both comparisons).

### *dsxF*^−/−^*XX* mutant ears have different mechanical tuning properties to *dsxF*^*+/+*^*XY* and *dsxF*^*+/+*^*XX* ears

**Figure 2:**
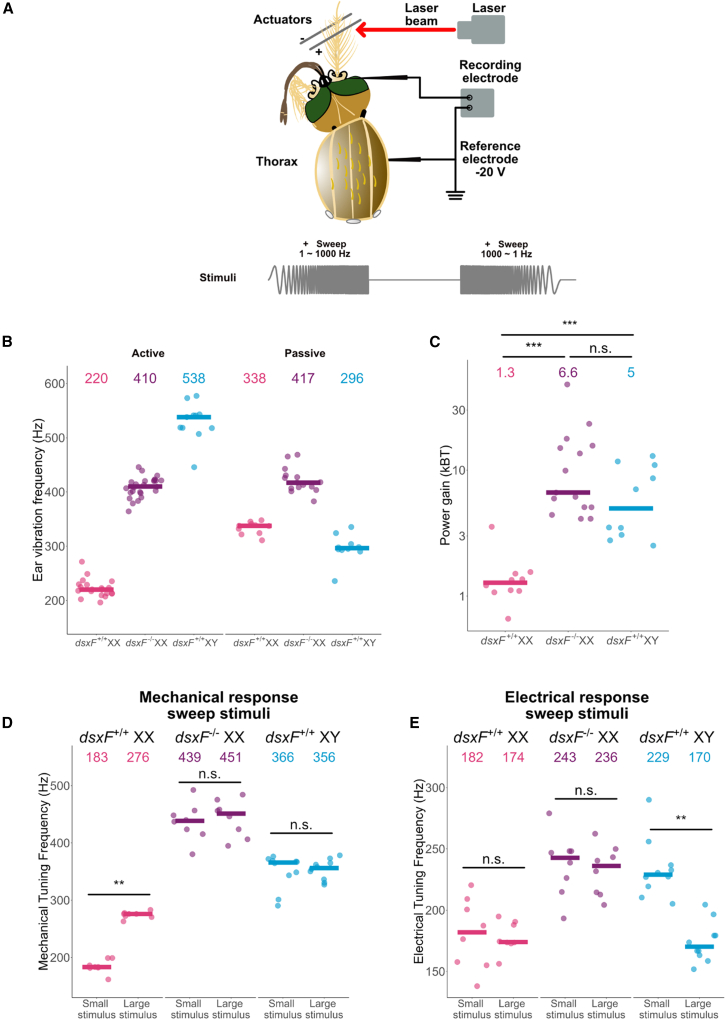
*dsxF*^−/−^*XX* ears show differences in frequency tuning in both active and passive states compared with other *dsxF*^+/+^*XX* and *dsxF*^+/+^*XY* ears, as well as in electrical responses to stimulation (A) Experimental setup for laser Doppler vibrometry recordings. Laser beam is focused toward the tip of the mosquito flagellum. Free fluctuation experiments used only unstimulated recordings. Experiments using electrostatic stimulation required the use of electrodes and electrostatic actuators. (B) Calculated best mechanical tuning frequencies of mosquito flagellar receivers in active (left) and passive (right) conditions. Median values are represented by solid lines, which are printed for each group at the top of the panel. Individual points are best mechanical tuning frequencies for individual mosquitoes from each group. See also Table 1. Sample sizes (females = active/passive; males = active quiescent/passive): *dsxF*^+/+^ *XX* = 20/10; *dsxF*^−/−^ *XX* = 25/15; *dsxF*^+/+^ *XY* = 10/21. (C) Calculated power gain for mosquito flagellar receivers. Median values are represented by solid lines, which are printed for each group at the top of the panel. Individual points are best mechanical tuning frequencies for individual mosquitoes from each group. All *dsxF*^+/+^ *XY* were in the non-SSO (quiescent) state for recordings. Mann-Whitney tests with Bonferroni corrections used for comparisons. See also Table 1. Sample sizes: *dsxF*^+/+^ *XX* = 10; *dsxF*^−/−^ *XX* = 15; *dsxF*^+/+^ *XY* = 10. ∗∗∗*p* < 0.001; not significant (n.s.), *p* > 0.05. (D) Calculated best frequencies of mechanical responses to smallest and largest sweep stimuli for each genotype. Median values are represented by solid lines, which are printed for each group at the top of the panel. Individual points are best mechanical tuning frequencies for individual mosquitoes from each group. All *dsxF*^+/+^ *XY* were in the SSO state for recordings. Paired Wilcoxon tests used for comparisons. See also Table 2 and Figure S3. Sample sizes: *dsxF*^+/+^ *XX* = 10; *dsxF*^−/−^ *XX* = 15; *dsxF*^+/+^ *XY* = 10. ∗∗∗*p* < 0.001; ∗∗*p* < 0.01; not significant (n.s.), *p* > 0.05. (E) Calculated best frequencies of electrical responses to smallest (left) and largest (right) sweep stimuli for each genotype Median values are represented by solid lines, which are printed for each group at the top of the panel. Individual points are best mechanical tuning frequencies for individual mosquitoes from each group. All *dsxF*^+/+^ *XY* were in the SSO state for recordings. Paired Wilcoxon tests used for comparisons. See also Table 2 and Figure S3. Sample sizes: *dsxF*^+/+^ *XX* = 10; *dsxF*^−/−^ *XX* = 15; *dsxF*^+/+^ *XY* = 10. ∗∗*p* < 0.01; not significant (n.s.), *p* > 0.05.

Given the sizable differences found in JO neuroanatomy, we next tested how these changes affected auditory function using laser Doppler vibrometry (Figure 2A). We investigated differences in frequency tuning and amplification in *dsxF*^−/−^ *XX* mosquito auditory systems when compared with *dsxF*^+/+^ *XX* and *dsxF*^+/+^ *XY* mosquitoes.

In the active state, *dsxF*^−/−^ *XX* mutants were found to be tuned to much higher frequencies than *dsxF*^+/+^ *XX* mosquitoes, with median frequencies of ∼410 Hz when compared with 220 Hz (Mann-Whitney test with Bonferroni correction; *p* = 3.597 × 10^−8^; Table 1; Figure 2B). *dsxF*^−/−^ *XX* frequency tuning was also found to be significantly different from quiescent and SSO *dsxF*^+/+^ *XY* individuals (Mann-Whitney tests with Bonferroni corrections; *p* = 8.229 × 10^−6^ and *p* = 1.386 × 10^−7^, respectively). Indeed, a frequency tuning of just over 400 Hz places *dsxF*^−/−^ *XX* mutants between *dsxF*^+/+^ *XX* (∼220 Hz) and non-SSO (quiescent) *dsxF*^+/+^ *XY* (∼520 Hz) frequency tunings (Table 1).

**Table 1:** Parameters obtained from free fluctuation analyses for all groups

	*dsxF*^+/+^*XX*	*dsxF*^−/−^*XX*	*dsxF*^+/+^*XY*
Sample size (active QUI)	20	25	11
Free fluctuation BF (Hz)	220.03 (3.76)	409.75 (3.78)	537.59 (10.65)
Q	1.19 (0.06)	23.50 (2.66)	12.08 (0.93)
Sample size (active SSO)	–	–	21
Free fluctuation BF (Hz)	–	–	352.79 (3.37)
Q	–	–	2,940.38 (1,047.10)
Sample size (Passive)	10	15	21
Free fluctuation BF (Hz)	337.52 (3.62)	417.22 (6.00)	303.55 (5.23)
Q	0.69 (0.01)	2.15 (0.11)	1.36 (0.07)
Apparent antennal mass (ng)	77.45 (3.04)	124.55 (9.53)	122.94 (4.87)
Sample size (Energy gain QUI)	10	15	10
Energy gain QUI (k_B_T)	1.28 (0.25)	6.64 (2.98)	4.97 (1.30)
Sample size (Energy gain SSO)	–	–	11
Energy gain SSO (k_B_T)	–	–	110,111.30 (52,581.120)

Median values from free fluctuation fits to data obtained from *dsxF*^*+/+*^*XX* and *XY* mosquitoes as well as *dsxF*^−/−^*XX* mosquitoes, with SEM values provided in brackets. *XX* analyses include active and passive states, whereas *XY* active analyses divide males into either quiescent (QUI) or self-sustained oscillation (SSO) states. No significant differences between the fits for the passive state in QUI and SSO males were found for any parameter (Mann-Whitney tests; *p* > 0.05), so the results were combined. Parameters include the best frequency of the flagellum, tuning sharpness (Q), apparent antennal mass, and estimated power gain for each group

Notably, these differences extended into the passive state: whereas all other lines were found to have significant differences between active and passive states in terms of frequency tuning (Mann-Whitney tests; *p* = 3.579 × 10^−5^ and *p* = 0.0003669 for *dsxF*^+/+^ *XX* and *dsxF*^+/+^ *XY*, respectively), no such differences were found for *dsxF*^−/−^ *XX* mutants (Mann-Whitney test; *p* = 0.4155).

*dsxF*^−/−^ *XX* mosquitoes also displayed significantly sharper frequency tuning, reflected in increased Q values, in the active state than any other lines (Mann-Whitney tests with Bonferroni corrections; *p* = 3.609 × 10^−8^ and *p* = 0.0003591 for comparisons with *dsxF*^+/+^ *XX* and *dsxF*^+/+^ *XY*, respectively).

In terms of calculated energy gains in the quiescent state, *dsxF*^−/−^ *XX* mutants displayed a masculine profile (Figure 2C), with energy gains similar to those exhibited by *dsxF*^+/+^ *XY* and significantly greater than those exhibited by *dsxF*^+/+^ *XX* (Mann-Whitney tests with Bonferroni corrections; *p* = 0.24 for *dsxF*^+/+^ *XY* compared with *dsxF*^−/−^ *XX*; *p* = 1.836 × 10^−6^ for *dsxF*^−/−^ *XX* compared with *dsxF*^+/+^ *XX*; *p* = 0.0006171 for *dsxF*^*+/+*^ *XY* compared with *dsxF*^+/+^ *XX*).

Importantly, *dsxF*^+/+^ *XY* exhibited SSOs, a masculine trait never observed for either type of *XX* mosquito. SSOs were of lower frequencies than the mechanical tuning best frequencies of non-SSO (quiescent) *dsxF*^+/+^ *XY* individuals; the energy gain of SSO-ing *dsxF*^+/+^ *XY* flagellar ears was several thousand times greater than those calculated for any *dsxF*^−/−^ *XX* mutant mosquito (Figures S2B and S2C).

### *dsxF*^−/−^*XX* mutant ears have different electrical tuning properties to *dsxF*^*+/+*^*XX* and *XY* ears

We next used established electrostatic stimulation paradigms to combine our investigations into ear mechanical tuning with electrophysiological recordings focused on the frequency tuning of the auditory nerve.^9^ By inserting an electrode into the antennal nerve, we were able to measure compound action potentials (CAPs) generated in response to electrostatic deflections of the mosquito flagellum. These deflections were induced by two stimuli types: force steps or pure tone sweeps.

First, we used step stimulation to investigate mechanical signatures of auditory transducer gating in both *XX* groups. *dsxF*^−/−^ *XX* mutant stiffness values were far greater than those for *dsxF*^*+/+*^ *XX* individuals (Figure S3A; Table S1) and indeed greater than all previously reported mosquito stiffness values, including for males.^9^ *dsxF*^−/−^ *XX* mutants also showed significantly greater CAP responses than *dsxF*^+/+^ *XX* for equivalent displacements (Figure S3B). Once these displacements were translated to the force domain, the significant extent of the increase in stiffness became apparent, however, with greater forces required for *dsxF*^−/−^ *XX* mutants to elicit comparable CAPs to *dsxF*^+/+^ *XX* (Figure S3C).

The use of frequency sweeps, pure tones that linearly change in frequency from 1 to 1,000 or 1,000 to 1 Hz, facilitated investigation into peak mechanical and electrical tuning frequencies, that is, the stimulation frequencies at which the maximal flagellar vibrations and CAP responses were identified. Previous investigations found differences in female mechanical tuning frequencies across different stimulation amplitudes^9^; by changing the intensity of the stimulus, we were able to test for frequency differences in both contexts across a range of stimulus amplitudes.

All *dsxF*^*+/+*^ *XY* mosquitoes exhibited SSOs throughout stimulus presentation. The mechanical best frequency of these SSO-ing *dsxF*^*+/+*^ *XY* mosquitoes did not change significantly when comparing the smallest to the largest stimulus intensities (paired Wilcoxon test; *p* = 1; Table 2; Figure 2D), with best frequencies remaining between ∼345 and ∼365 Hz across the entire range of stimulus intensities tested (Figure S3D). For *dsxF*^*+/+*^ *XX* mosquitoes, however, we saw an increase in best frequency from ∼190 to ∼275 Hz from the smallest to the largest intensity, in agreement with previous reports^9^ of such a shift for white noise stimulation (paired Wilcoxon test; *p* = 0.007813; Figures 2D and S3D). Similar to *dsxF*^*+/+*^ *XY*, the mechanical best frequency of *dsxF*^−/−^ *XX* mutants did not change significantly when comparing the smallest to the largest stimulus intensities (439 vs. 451 Hz, respectively; paired Wilcoxon test; *p* = 0.1953; Table 2; Figure 2D). *dsxF*^−/−^ *XX* mosquitoes maintained a mechanical best frequency near ∼445 Hz across the entire range of stimulus intensities (Figure S3D).

**Table 2:** Parameter values for each group for mechanical and electrical responses to sweep stimulation

	*dsxF*^+/+^*XX*	*dsxF*^−/−^*XX*	*dsxF*^+/+^*XY*
Sample size	8	8	10
Mechanical BF largest sweep (Hz)	276.04 (2.09)	451.15 (11.33)	356.26 (5.54)
Mechanical BF smallest sweep (Hz)	183.45 (4.15)	438.54 (11.53)	365.75 (9.61)
Electrical BF largest sweep (Hz)	174.03 (4.42)	236.08 (7.18)	170.25 (5.21)
Electrical BF smallest sweep (Hz)	181.85 (10.23)	242.78 (9.09)	228.95 (7.71)
Displacement gain	2.87 (0.15)	1.38 (0.11)	13.95 (0.97)

Median values from analysis of mechanical and electrical responses to sweep stimulation in *dsxF*^*+/+*^*XX* and *XY* mosquitoes as well as *dsxF*^−/−^*XX* mosquitoes, with SEM values provided in brackets. BF, Best Frequency.

On the other hand, the electrical best frequency of *dsxF*^+/+^ *XY* mosquitoes was found to decrease from ∼230 to ∼180 Hz between the smallest and largest stimulus intensities, a phenomenon not observed for *dsxF*^+/+^ *XX* mosquitoes (paired Wilcoxon tests; *p* = 0.001953 and *p* = 0.7792, respectively; Table 2; Figure 2E). Similarly to control females, the electrical best frequency of *dsxF*^−/−^ *XX* mutants did not change from the smallest to the largest intensity presentations (paired Wilcoxon tests; *p* = 1; Table 2; Figure 2E). For these mutants, the electrical best frequency remained at ∼240 Hz across the entire range of stimulus intensities tested (Figure S3D). In short, *dsxF*^+/+^ *XX* (female) nerves display only one uniform frequency tuning of ∼180 Hz for both small and large stimulus intensities. *dsxF*^+/+^ *XY* (male) nerves, in turn, show two tuning regimes: for large stimulus intensities, responses peak around ∼180 Hz, resembling the tuning of female nerves, but for small stimulus intensities, there is an additional higher-frequency tuning around ∼230 Hz. The nerve responses of *dsxF*^−/−^ *XX* (intersex) females, finally, show the male-specific higher-frequency tuning at all stimulus intensities.

Changes in *dsxF*^+/+^ *XX* mechanical best frequency occurred in an almost step-like fashion once the stimulus force reached a specific threshold (Figure S3D). On the other hand, the shift in electrical best frequency observed for all male mosquitoes appeared more gradual, with a steady decrease in frequency for- increasingly larger stimuli (Figure S3D).

We then calculated the displacement over force ratio for each stimulus intensity per individual mosquito and used these values to estimate the displacement gain for each genotype (Table 2). In contrast to the previous estimates of energy gain, we here found that *dsxF*^−/−^ *XX* mutants had significantly lower displacement gains than all other groups (Welch t tests with Bonferroni corrections; *p* = 1.382 × 10^−6^ and *p* = 2.356 × 10^−5^ for comparisons with *dsxF*^+/+^ *XY* and *dsxF*^+/+^ *XX*, respectively). This finding is consistent with the vast increase in flagellar stiffness observed in intersex mosquitoes (Figure S3A; Table S1), which overshoots the male wild-type (*dsxF*^+/+^ *XY*) condition. Furthermore, *dsxF*^+/+^ *XY* mosquito displacement gain values were significantly greater than *dsxF*^+/+^ *XX* (Welch t test with Bonferroni correction; *p* = 3.339 × 10^−6^).

### RNA-seq analyses of mosquito ears identified differences in expression of numerous ciliary motility–related factor between groups

The above-mentioned experiments focused on anatomical and functional tests of hearing, demonstrating sizable differences between *dsxF*^−/−^ *XX* and control mosquitoes of both sexes, with several mutant characteristics shifting toward the *dsxF*^+/+^ *XY* mosquito phenotype. We investigated these differences at the transcriptional level to identify molecular factors that could account for the traits of “auditory maleness.”

**Figure 3:**
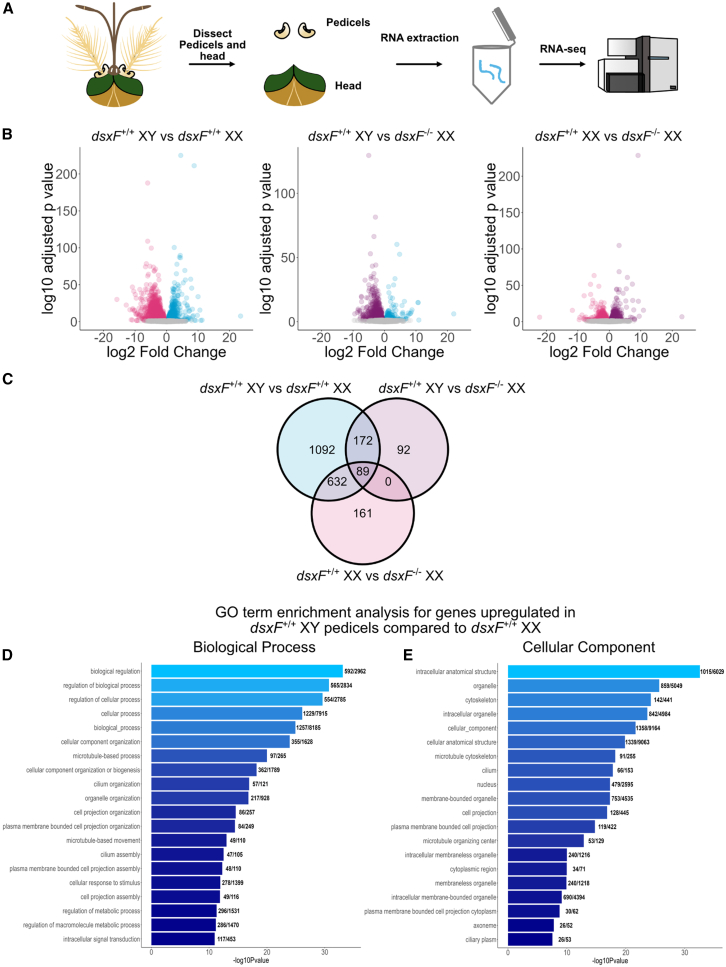
Significant enrichment of ciliary motility–related genes in *dsxF*^+/+^ *XY* and *dsxF*^−/−^ *XX* ears compared with *dsxF*^+/+^ *XX* (A) Schematic of experimental assay, including tissue dissection. (B) Volcano plots of differential gene expression between (left) *dsxF*^+/+^ *XY* and *dsxF*^+/+^ *XX* pedicels, (middle) *dsxF*^+/+^ *XY* and *dsxF*^−/−^ *XX* pedicels, and (right) *dsxF*^−/−^ *XX* and *dsxF*^+/+^ *XX* pedicels. (C) Venn diagram of pedicel gene intersections showing those differentially regulated between groups. Upregulated group is to the left-hand side of the group name. (D and E) GO term enrichment analysis for genes upregulated in *dsxF*^+/+^ *XY* pedicels compared with *dsxF*^+/+^ *XX* (Table S3). (D) Biological process. (E) Cellular component.

Given previous reports of peaks in male and female *An. gambiae* activity at dusk,^12^ we collected *dsxF*^+/+^ *XX* and *dsxF*^+/+^ *XY*, as well as and *dsxF*^−/−^ *XX*, mosquitoes at the onset of dusk, dissected the pedicels of these mosquitoes, and submitted them for RNA-seq analysis (Figure 3A). The following comparisons were conducted for differential gene expression:

*dsxF*^+/+^ *XX* vs. *dsxF*^+/+^ *XY* pedicels (M*v*F) (3,353 differentially expressed genes)
*dsxF*^+/+^ *XX* vs. *dsxF*^−/−^ *XX* pedicels (I*v*F) (2,029 differentially expressed genes)
*dsxF*^−/−^ *XX* vs. *dsxF*^+/+^ *XY* pedicels (M*v*I) (1,332 differentially expressed genes)

These comparisons are visualized via volcano plots (Figure 3B) and a Venn diagram (Figure 3C). Raw count data are provided in Table S2. To explore the molecular underpinnings of auditory maleness, from the 3,353 differentially expressed genes in the *dsxF*^+/+^ *XY* vs. *dsxF*^+/+^ *XX* (denoted M*v*F) comparison, we focused our primary analyses on the 1,985 genes found to be significantly upregulated in male pedicels when compared with females. We used the *dsxF*^−/−^ *XX* vs. *dsxF*^+/+^ *XX* (denoted I*v*F) and *dsxF*^+/+^ *XY* vs. *dsxF*^−/−^ *XX* (denoted M*v*I) comparisons to create subsets of the 1,985 genes upregulated in the M*v*F comparison. Of the 1,985 genes, 632 (subset *s*_*632*_) were found to be upregulated in both *dsxF*^+/+^ *XY* and *dsxF*^−/−^ *XX* compared with *dsxF*^+/+^ *XX* but were not differentially expressed between *dsxF*^+/+^ *XY* and *dsxF*^−/−^ *XX*. Thus, the subset contains genes highly expressed in *dsxF*^+/+^ *XY* compared with *dsxF*^+/+^ *XX* that also exhibit full recovery of male expression in *dsxF*^−/−^ *XX*.

A total of 172 genes (subset *s*_*172*_) were found to be upregulated in *dsxF*^+/+^ *XY* compared with *dsxF*^+/+^ *XX* and *dsxF*^−/−^ *XX*, but not differentially expressed between the *dsxF*^−/−^ *XX* and *dsxF*^+/+^ *XX* genotypes. These genes thus exhibit higher expression in *dsxF*^+/+^ *XY* and are not under the influence of the DsxF mutation. Of the remaining genes, 89 (subset *s*_*89*_) were found to be upregulated in *dsxF*^+/+^ *XY* compared with both *dsxF*^+/+^ *XX* and *dsxF*^−/−^ *XX*, but were also upregulated in *dsxF*^−/−^ *XX* compared with *dsxF*^+/+^ *XX*. Thus, this subset represents transcripts highly expressed in *dsxF*^+/+^ *XY* in which *dsxF*^+/+^ *XY* expression has been recovered only partly in the *dsxF*^−/−^ *XX* group.

Finally, the last 1,092 genes (subset *s*_*1092*_) of the 1,985 set were found to be upregulated in *dsxF*^+/+^ *XY* compared with *dsxF*^+/+^ *XX* but neither in *dsxF*^+/+^ *XY* compared with *dsxF*^−/−^ *XX* nor in *dsxF*^−/−^ *XX* compared with *dsxF*^+/+^ *XX*. This large subset includes genes that, on average, showed increased expression in *dsxF*^−/−^ *XX* compared with *dsxF*^*+/+*^ *XX* but not enough to yield statistically significant results. Subsets of the 1,985 genes are shown in Figures 3C and S4 includes visual comparisons of the log median expression for each genotype within each subset to aid understanding of their interpretations. Full gene lists for each subgroup are included in Table S3. Figure S5 includes analyses of genes upregulated in *dsxF*^+/+^ *XX* and *dsxF*^−/−^ *XX* compared with *dsxF*^+/+^ *XY*.

These subsets thus facilitate informed explorations into DsxF- and non-DsxF-inhibited auditory maleness and also inferences on the molecular basis of our experimental findings. The transcripts of *s*_*632*_ constitute the transcriptomic basis of auditory maleness under the inhibition of DsxF, whereas those of *s*_*89*_ reflect those aspects of auditory maleness only partly influenced by it; transcripts of *s*_*1092*_ may also reflect these components at a level non-differentiable by statistical analysis. Finally, the transcripts of *s*_*172*_ constitute auditory maleness that is not under the regulation of DsxF.

We next conducted global, GO enrichment and gene-specific annotation analyses independently on each of the three sets to investigate their functional biological characteristics. Selected elements of GO enrichment analyses are included in Table S4, and their expression levels visualized in Figures 3D and 3E.

GO analyses on *s*_*632*_ revealed several terms relating to the structure and organization of microtubules, cilia, and the motor complex. These include microtubular structural components such as AGAP008275-RA (tektin-2); AGAP000219-RA (tektin-3); AGAP001227-RA (gamma-tubulin complex component 5); AGAP010971-RA (tubulin); AGAP002334-RA (spastin), which functions as a microtubule severing protein; AGAP006324-RA (*nompB*), which is required for the assembly of sensory cilia; and AGAP005250-RA (protein kintoun) and AGAP009594-RA, which are dynein assembly factors. Other proteins related to microtubule and ciliary structure and regulation highlighted by our analyses are AGAP012726-RA and AGAP003120-RA, which are stabilizers of axonemal microtubules; AGAP013430-RA (piercer of microtubule wall); and AGAP006735-RA and AGAP008870-RA (cilia and flagella associated proteins). Thus, at least partly, DsxF-inhibited auditory maleness seems to involve setting up the structures used by motor proteins to generate motion. In terms of transcripts encoding motor proteins, *s*_*632*_ included some kinesins (AGAP007592-RA, AGAP002427-RA, AGAP000561-RA) and dyneins (AGAP011441-RA, AGAP000320-RA, AGAP010165-RA, AGAP009568-RA, AGAP004416-RA).

We saw previously that both *dsxF*^*+/+*^ *XY* and *dsxF*^−/−^ *XX* mosquitoes exhibited similar power gains that were also greater than *dsxF*^*+/+*^ *XX* mosquito estimates; the shared structural and motor elements just discussed may directly relate to this finding. We also saw that contrary to *dsxF*^*+/+*^ *XX* mosquitoes, both *dsxF*^*+/+*^ *XY* and *dsxF*^−/−^ *XX* mosquitoes exhibited mechanical frequency tuning that was largely invariant to stimulus intensity. Set *s*_*632*_ includes elements related to ion channels, receptors, and signaling that could be involved in tuning the flagellum. The set includes, for example, *nanchung* (AGAP012241-RA), a key element of the mechanotransduction complex. Further elements of *s*_*632*_ include *AgOctβ2R* (AGAP002886-RA), an octopamine receptor that has recently been shown to influence male ear tuning,^12^ a piezo-type mechanosensitive ion channel (AGAP009276-RB), some chloride ion channels (AGAP005599.R500, AGAP005599.R502, and AGAP009616-RA), some potassium ion channels (AGAP001281-RB, AGAP003709-RC, AGAP003709-RD), a calcium ion channel (AGAP008028-RA), and a G protein–coupled receptor (AGAP001562-RA). Finally, several cuticle-related transcripts (AGAP000986-RA, AGAP000988-RA, AGAP006830-RA, AGAP006828-RA, AGAP009868-RA, AGAP006283-RB, and AGAP012487-RA) were included in *s*_*632*_ and could be involved in the male-like enlargement of the JO observed in *dsxF*^−/−^ *XX* mutants.

As alluded to above, transcripts related to auditory maleness only partly influenced by DsxF are included in *s*_*89*_, and possibly also *s*_*1092*_. Several ion channels, receptors, and signaling-related transcripts that were only partly recovered in the *dsxF*^−/−^ *XX* mutants relative to *dsxF*^*+/+*^ *XY* are found in *s*_*1092*_, including two potassium ion channels (AGAP000254-RA and AGAP011924-RA), the GABA-gated chloride ion channel *Rdl* (AGAP006028-RC) and three other chloride ion channels (AGAP005599.R499, AGAP005599.R504, and AGAP005777-RA), *TRPM* (AGAP006825-RA), a nicotinic acetylcholine receptor component (AGAP002152-RA), two ionotropic receptors (AGAP001478-RA and AGAP005527-RA), and two G protein–coupled receptors (AGAP002888-RC and AGAP004222-RA).

Set *s*_*89*_ includes just two transcripts encoding dynein motor proteins (AGAP008689-RB and AGAP011540-RA) that were only partly recovered in *dsxF*^−/−^ *XX* mutants relative to *dsxF*^*+/+*^ *XY*, whereas set *s*_*1092*_ includes two dyneins (AGAP006887-RA and AGAP004030-RA), two kinesins (AGAP000159-RA and AGAP000575-RA), and four myosins (AGAP004403-RA, AGAP000776-RA, AGAP029989.R839, and AGAP011138-RB). A characteristic male feature that was not recovered in *dsxF*^−/−^ *XX* was that of SSOs of the flagellum. It is possible that partial recovery of expression in the signaling and motor-related transcripts just discussed is related to the lack of SSOs in the *dsxF*^−/−^ *XX* mutants. These transcripts, thus, may form part of the machinery that brings SSOs about.

It is also possible that SSOs are a manifestation of auditory maleness that is not under the influence of DsxF. Looking at *s*_*172*_, we find several ion channels, receptors, and signaling transcripts that are significantly higher in *dsxF*^*+/+*^ *XY* compared with both *dsxF*^*+/+*^ *XX* and *dsxF*^−/−^ *XX* but similar in expression in *dsxF*^*+/+*^ *XX* and *dsxF*^−/−^ *XX*. Such transcripts could be key factors for SSOs, as these flagellar oscillations have been shown to be influenced by neurochemical and efferent-related modulation of the male hearing system.^9^^,^^12^ The set includes two potassium ion channels (AGAP001284-RA, AGAP005251-RA), a histamine-gated chloride ion channel subunit (AGAP001990-RA), a nicotinic acetylcholine receptor subunit (AGAP005034-RA), and a metabotropic glutamate receptor (AGAP005034-RA).

To provide additional support for our comparisons between *dsxF*^*+/+*^ *XY* and *dsxF*^*+/+*^ *X*X, we reanalyzed previously published RNA-seq pedicel data for *An. gambiae* (Kisumu strain).^14^ Using the same analysis method as for the *dsxF* mosquitoes, we found 1,447 genes significantly upregulated in male Kisumu pedicels compared with female Kisumu and 2,073 significantly downregulated (Figure S6A). By comparing the lists of significantly upregulated and downregulated genes in both analyses, we found 1,568 genes significantly upregulated in male compared with female pedicels in both strains (44.3% of all differentially expressed genes), as well as 2,051 significantly downregulated (Figure S6B and S6C). GO term enrichment analyses of the 1,568 genes significantly upregulated in both strains identified terms related to microtubules, cytoskeleton, and cilium (Figure S6D; Table S5). Of the 82 genes highlighted in Table S3 that were upregulated in *dsxF*^*+/+*^ *XY* pedicels compared with *dsxF*^*+/+*^ *X*X, 66 were also upregulated in male Kisumu pedicels compared with females.

### Comparison with previously published testes RNA-seq data highlights tissue-specific factors related to the motile cilia machinery

**Figure 4:**
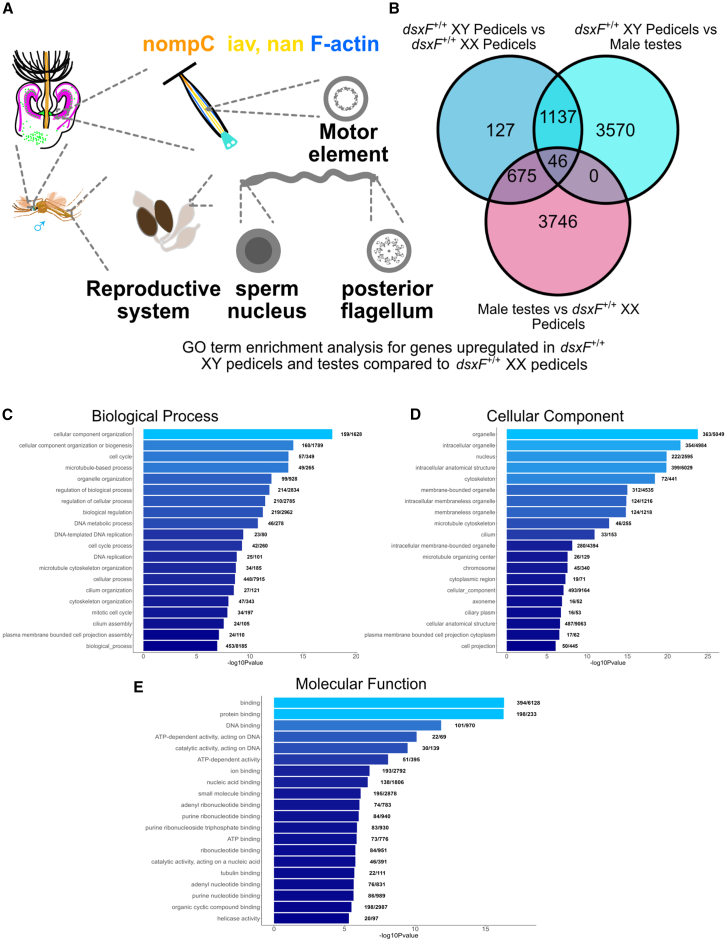
Comparisons of ears and testes enables identification of tissue-specific ciliary motility–related factors (A) Schematic of tissues containing motile cilia in male mosquitoes. (B) Venn diagram of pedicel/testes gene intersections showing those differentially regulated between groups. Upregulated group is to the left-hand side of the group name. (C) GO term enrichment analysis (biological process terms) for genes upregulated in male pedicels and testes compared with *dsxF*^+/+^ *XX*. (D) GO term enrichment analysis (cellular component terms) for genes upregulated in male pedicels and testes compared with *dsxF*^+/+^ *XX*. (E) GO term enrichment analysis (molecular function terms) for genes upregulated in male pedicels and testes compared with *dsxF*^+/+^ *XX*.

The presence of SSOs in male mosquitoes constitutes complementary evidence for their “active hearing,” the broader phenomenon by which the ear injects energy into and thereby amplifies the reception of external signals. Several motor protein and motor protein–related transcripts were identified in the previous paragraphs that characterize both DsxF-inhibited and DsxF-independent male-specific expression, suggesting a more fundamental link between auditory cellular motility and maleness as auditory neurons and sperm are the only two known specialized cells that contain motile cilia in insects (Figure 4A).

To investigate this relationship further we contrasted our pedicel RNA-seq dataset with an existing testes RNA-seq database.^27^ We identified 675 genes (*s*_*675*_) upregulated in both male pedicels and testes compared with *dsxF*^+/+^ *XX* pedicels (Figure 4B). GO enrichment analyses applied to *s*_*675*_ genes identified terms related to dynein assembly and microtubule binding/regulation (Figures 4C–4E). These dynein- and microtubule-related genes could also be differentially regulated between male tissues, with some showing significantly increased expression only in pedicels or testes (Table S6). Further analyses showed that, similar to *s*_*632*_ of the pedicel comparisons’ intersectional subsets, *s*_*675*_ included transcripts AGAP005250-RA (protein kintoun), AGAP010971-RA (tubulin), and AGAP008275-RA (tektin-2).

Furthermore, *s*_*675*_ included two ionotropic receptors also found in *s*_*1092*_ (AGAP005527-RA and AGAP001478-RA), a potassium ion channel (AGAP003709-RA), and a piezo-type mechanosensitive ion channel component (AGAP009276-RB) that were also in *s*_*632*_. In terms of motor-related transcripts, *s*_*675*_ included one dynein (AGAP004030-RA), two myosins (AGAP004403-RA and AGAP01138-RA), and one kinesin (AGAP000575-RA) that were also in *s*_*1092*_ (with the exception of AGAP01138-RA; *s*_*1092*_ included the -RB isoform of the transcript). Set *s*_*675*_ also included four additional dyneins that were in *s*_*632*_ (AGAP004416-RA, AGAP011441-RA, AGAP009568-RA, and AGAP010165-RA).

## Discussion

### Disruption of the female-specific exon of *doublesex* leads to female hearing systems being partially converted to male hearing systems

Sexual dimorphisms in mosquito hearing function are in part the result of differences in ear anatomy. The roles played by different neuronal populations as well as auditory efferents are a major part of these differences in function.^1^ Auditory efferent fibers, although clearly abundant in the ears of male *An. gambiae*, were found in far smaller numbers for conspecific females both here and in previous work.^9^ The loss of the *dsxF* protein in homozygous mutant females appeared to not only increase the number of neurons in mutant female ears compared with wild-type females but also increase the number of efferent terminals located in the JO (Figures 1 and S1). This mutation was not sufficient, however, to completely transform female ears into male ears, with *dsxF*^−/−^ *XX* ears remaining smaller than all male ears in terms of neuronal counts. This seemingly intersex number of neurons in *dsxF*^−/−^ *XX* JOs could be related to the increased magnitude of the compound action potentials we recorded in *dsxF*^−/−^ *XX* compared with *dsxF*^+/+^ *XX.*

Although absolute changes in neuronal number are important, the number of cuticular prongs, which mechanically connect JO neurons to the base of the flagellum, are also relevant. These prongs physically partition neuronal groups into distinct functional planes.^9^ Further work is needed not only to quantify these prongs—likely to show intersex characteristics in *dsxF*^−/−^ *XX* ears given previously reported sex-related differences^25^—but also to count the neurons present within the different JOs. The incomplete transformation of female ears observed after disrupting *dsxF* expression is presumably due to the absence of DsxM expression, which appears necessary (along with *fruitless*) for full masculinization.

Functionally, *dsxF*^−/−^ *XX* mutant mechanical and electrical tuning appeared different from controls of both sexes (Figures 2 and S2). Mechanical frequency tuning was distinct from both control males and females (Table 1). *dsxF*^−/−^ *XX* mutants were able to produce significantly greater compound action potentials than *dsxF*^+/+^ *XX* following exposure to identical stimuli in terms of flagellar displacement, in agreement with their apparent increase in neuronal number. Interestingly, the *dsxF*^−/−^ *XX* ear was also found to be much stiffer than ears of *dsxF*^+/+^ *XX*, with substantially greater amounts of force required to achieve equivalent displacements (Figure S3). Neurons have been shown to contribute to the stiffness of the antennal sound receiver in *Drosophila*^28^; the increase of neuronal number in *dsxF*^−/−^ *XX* ears is thus a contributing cause for the increased CAP responses and stiffness, but other causal factors are also likely to be at play. Increases in stiffness could have several underlying causes, from changes in ion channel expression^29^ to different numbers of mechanotransduction units to altered cuticular structures attaching JO neurons to the flagellum^30^^,^^31^ (Table S3). Most notably in this context, another principal parameter of receiver mechanics, the apparent mass of the *dsxF*^−/−^ *XX* flagellum is fully restored to the level of the *dsxF*^+/+^ *XY* controls.

### SSOs remain a male-specific phenomenon

Perhaps most importantly, however, mutants failed to exhibit SSOs, the hallmark of male mosquito ears and potentially the central component of the male’s ability to identify the faint sound of a female within the din of a swarm.^9^^,^^13^^,^^14^ Autonomous receiver vibrations have been observed in females following injection of DMSO,^13^ but these are quantitatively and qualitatively distinct from the SSOs seen in male receivers and much more resemble the autonomous oscillations observed in *Drosophila* after genetic or pharmacological manipulation. Most notably, injection of compounds that specifically sever the efferent/afferent feedback loops between the ear and brain induced SSOs only in males.^9^ Our data—and our corresponding intersectional analysis—will help to close in on the molecular machinery for SSOs. Although it is possible, and even likely, that SSOs originate from the interaction of several molecular factors, our study has made a dedicated effort to narrow the list of candidates down to a manageable number. The most promising list is provided by subset *s*_*172*_.

The substantial amplification (lifting the receiver’s power gain by almost four orders of magnitude) provided by the male’s SSOs is a remarkable feature,^9^ rivaling the unique amplification carried out by the piezo-crystal-like protein Prestin in the lateral walls of mammalian outer hair cells.^32^ Although it is likely that SSOs originate from ciliary motility factors, such as dyneins, and although it has also been shown that the auditory power gain in *Drosophila* is independent of Prestin homologs,^33^ it remains to be seen if this also holds true for mosquitoes. Although the precise molecular causes of SSOs are thus not yet resolved, it seems clear that SSOs are a property originating from the auditory JO neurons proper, although they may be modulated by efferent, neuromuscular, or other synaptic input.^34^

### Transcriptomic analyses revealed upregulation of ciliary motility factors in the absence of DsxF

Our RNA-seq analyses found upregulation of a number of ion channels and receptors in *dsxF*^+/+^ *XY* pedicels when compared with *dsxF*^+/+^ *XX*. Any one, or combination, of these candidates may be necessary for SSOs. Beyond changes in ion channel counts, however, our analyses also found substantial enrichment of motile ciliary-related factors in *dsxF*^+/+^ *XY* compared with *dsxF*^+/+^ *XX* pedicels, some of which were fully recovered upon DsxF mutation and some only partly—this extended to microtubules, dyneins, myosins, kinesins, and flagella-associated proteins. Importantly, many of these crucial ion channels, receptors, and ciliary motility factors have been reported to be part of a mechanotransduction machinery regulating the active hearing properties of *Drosophila melanogaster*.^35^^,^^36^

The upregulation of gene expression related to ciliary motility and ion channels and receptors in *dsxF*^−/−^ *XX* compared with *dsxF*^+/+^ *XX* could be due to (1) increase in the expression of genes related to mechanotransduction reflecting an increase in the number of JO neurons and thus the associated mechanotransduction units in *dsxF*^−/−^ *XX* ears compared with *dsxF*^+/+^ *XX*; (2) the transcriptional inhibitory role played by DsxF to repress the expression of these genes, so that in the absence of DsxF *dsxF*^−/−^ *XX* showed an increase in gene expression; or (3) a combination of these two possibilities. The first hypothesis would fit in the case of genes exhibiting intermediate expression in *dsxF*^−/−^ *XX*. On the other hand, the absence of DsxF in *dsxF*^−/−^ *XX* could result in full masculinization of gene expression despite an intermediate number of JO neurons in *dsxF*^−/−^ *XX,* supporting a transcriptional inhibitory role of DsxF. DsxF and its male counterpart, DsxM, share the same DNA-binding zinc finger domain, meaning they share the same downstream target genes.^37^ DsxF and DsxM, however, could exert different transcriptional effects on the same target genes (i.e., one acting as a transcriptional activator and the other as a transcriptional repressor, or vice versa) due to sex-specific differences at their C termini, although both likely form transcription factor complexes rather than acting independently.^38^^,^^39^^,^^40^^,^^41^ The opposite roles of DsxM and DsxF and their downstream target genes, in turn, drive a sex-specific developmental program to give rise to sexually dimorphic morphologies and tissue functions.^38^ For example, in *Drosophila*, *dsxM* has been reported to promote neuronal development, whereas *dsxF* promotes neuronal apoptosis.^41^

Interestingly, despite the absence of DsxM, *dsxF*^−/−^ *XX* mutants, which also lacked DsxF, were still able to promote the expression of genes related to ciliary motility (Table S3). This would also indicate that DsxM may not be implicated in the transcriptional activation of these genes and that lifting the inhibitory effect of DsxF is sufficient (along with other co-factors) to drive gene expression within the JO. Our results therefore suggest that mosquito JOs represent an ideal target to investigate the interactions between Dsx isoforms on downstream gene expression and future work focusing on elucidating the effects of DsxM in shaping mosquito ears could provide further clarification on how *dsx* shapes sexually dimorphic mosquito hearing.

The fundamental changes in the motile apparatus underlying active hearing function may offer new avenues to explore sexual dimorphisms in the fundamental ear machinery. Our findings highlight the developmental and mechanistic relationship between male ears and testes, with several motor proteins, and other cell motility–related elements, being co-expressed in the two tissues (Table S6). In particular, several motility-related transcripts that reached “masculine” (or toward masculine) levels of expression in the ears of *dsxF*^−/−^ *XX* mutants showed shared levels of expression between male ears and testes and had significantly higher expression than the respective transcripts in the female ear. This finding is also promising in terms of vector control interventions targeting reproduction as disruption of molecular elements expressed in both tissues could improve the potency of interventions by disrupting both hearing function and sperm motility.^35^^,^^42^ Prior work in *Drosophila melanogaster* has identified multiple shared factors in sperm and JO neurons, including the axonemal dynein machinery and assembly factors.^35^^,^^36^^,^^43^^,^^44^ Testing of mosquito orthologs of these genes could help identify novel targets for mosquito control that could simultaneously inhibit male mating and hearing.

### Investigating the sex determination pathway facilitates identification of novel targets for vector control

*dsx* represents only one component of the mosquito sex determination pathway, with *femaleless, SOA,* and *yob* all playing separate roles.^16^^,^^45^^,^^46^^,^^47^ Considering that many of these lines have been highlighted for use in mosquito control programs, it is essential that their auditory function be first tested before release not only in *Anopheles* but also in other species. The mosquito ear represents a highly promising target for novel vector control methods, with the development of insecticidal resistance increasing the pressure to develop new control options.^5^^,^^48^ The release of gene drive–coupled hearing gene mutants could help collapse local populations in the same manner as predicted for the release of *dsx* mutants. Alternatively, strategies for improving male mating fitness by increasing their phonotaxis efficiency could also be explored to ensure that gene drive–based control methods are forced into their target gene pools, increasing the likelihood of population collapse.

The overwhelming reliance of males on hearing for the identification of conspecific females during courtship suggests that interfering with this process via pharmacological or acoustic tools holds great potential. However, acoustic lures targeting males thus far have failed to transfer their efficacy from the laboratory to field trials^49^; identifying the crucial component of this failure is challenging without first understanding the networks that underlie mosquito hearing.

### Limitations of the study

Here, we investigated only the effect of disrupting the female-specific isoform of *dsx* (*dsxF*); the role played in the sex determination pathway by the male-specific isoform (*dsxM*), therefore, remains unclear. We were unable to identify the precise molecular basis of male mosquito SSOs, although our gene lists provide guidance for future research. Finally, although *dsx* plays major roles during development, in this study we investigated only adult mosquito ear anatomy and gene expression; future work could explore the effect of DsxF mutation on the development of *An. gambiae* JOs.

## Resource availability

### Lead contact

Further information on all experiments conducted as part of this report, in addition to requests for resources, can be requested from the lead contact, Joerg T. Albert (joerg.albert@uni-oldenburg.de).

### Materials availability

This study did not generate new unique reagents.

### Data and code availability

• RNA-seq data are available via the GEO repository (GEO accession number: GSE289862).
• This paper does not report original code.
• Additional information regarding analysis protocols/data collection is available via from the lead contact upon request.

## Acknowledgments

The authors would like to thank Carla Siniscalchi (Imperial College London) for providing G3 strain pupae.

This work was supported by grants from the Biotechnology and Biological Sciences Research Council, UK (BBSRC, BB/V007866/1 to J.T.A.), The Human Frontier Science Program (HFSP grant RGP0033/2021 to J.T.A.), and the European Union’s Horizon 2020 research and innovation program (H2020-ERC-2014-CoG/648709/Clock Mechanics, to J.T.A.). M.P.S. and J.S. were supported by the European Research Council grant H2020-ERC-2014-CoG/648709, an ANTI-VeC pump-priming grant (AV/PP/0028/1, to J.T.A.), and a UCL GCRF small grant (QR GCRF 551039 to J.T.A.). M.G. was also supported by the UCL GCRF small grant and the ANTI-VeC pump-priming grant. M.P.S., Y.M.L., and Y.Y.J.X. were co-funded by a Tokai Pathways to Global Excellence (T-GEx), as part of the MEXT Strategic Professional Development Program for Young Researchers, grant (0121an0002) and an MEXT KAKENHI Grant-in-Aid for Research Activity Start-up (No. JP22K15159). M.A. received funding through a Marie Sklodowska-Curie Individual Fellowship from the European Commission (H2020-MSCA-IF-2016/752472). J.B. was supported by BB/V007866/1. J.T.A. was supported by the European Research Council grant H2020-ERC-2014-CoG/648709 and UCL GCRF, and ANTI-VeC grants. RNA-seq was provided via InfraVec2 (#5251).

## Author contributions

Conceptualization, M.P.S., M.G., M.A., J.S., K.K., A.C., and J.T.A.; methodology, M.P.S., M.G., Y.M.L., Y.Y.J.X., and J.T.A.; investigation, M.P.S., M.G., M.A., and J.B.; resources, M.P.S. and J.T.A.; writing, M.P.S., M.G., Y.M.L., and J.T.A.; funding acquisition, M.P.S. and J.T.A.; supervision, M.P.S. and J.T.A.

## Declaration of interests

The authors declare no competing interests.

## STAR★Methods

### Key resources table

**Table:** 

REAGENT or RESOURCE	SOURCE	IDENTIFIER
**Antibodies**		

Mouse monoclonal anti-SYNORF1 3C11	Developmental Studies Hybridoma Bank	RRID: AB_528479
Cy™3 AffiniPure™ Goat Anti-Horseradish Peroxidase	Jackson Immuno Research	RRID: AB_2338959
Phalloidin, Alexa Fluor™ 647	Thermo Fisher	# A22287; RRID:AB_2620155
Goat anti-mouse IgG Secondary Antibody, Alexa Fluor™ 488	Thermo Fisher	#A-11029; RRID: AB_2534088

**Deposited data**		

RNAsequencing data	This paper	GEO: GSE289862

**Experimental models: Organisms/strains**		

*Anopheles gambiae* (G3 strain)	Kyrou et al.^15^	N/A
*Anopheles gambiae* (*doublesexF* mutant strain)	Kyrou et al.^15^	N/A

**Software and algorithms**		

Axiovision (version 4.3)	Carl Zeiss Microscopy, LLC	https://www.micro-shop.zeiss.com
ImageJ (version 1.54g)	ImageJ	https://imagej.net/ij/
MATLAB (version 2021a)	MathWorks	https://www.mathworks.com
R (version 4.3.1)	R Core Team	https://www.R-project.org/
Sigmaplot (version 9)	Systat Software, Inc	https://grafiti.com/sigmaplot-detail/
Spike2 (version 10.08)	Cambridge Electronic Design	https://ced.co.uk/products/spkovin
VibSoft (version 6.0)	Polytec	https://www.polytec.com

### Experimental model and study participant details

#### Mosquitoes

*An. gambiae* G3 strain (*dsxF*^+/+^) and *dsxF*^−/−^ *XX* mutant pupae were reared by the Crisanti lab at Imperial College London. *dsxF*^+/+^ pupae were sex separated and kept in single sex cages in incubators maintained at 27°C and 60–70% relative humidity using a 12 h:12 h light/dark cycle. *dsxF*^−/−^ pupae were not sex separated but were otherwise reared in identical conditions. Mosquitoes were supplied with a constant source of 10% glucose solution. Cow blood feeding, when required, was conducted by a trained research assistant using a Hemotek system (Discovery Workshops, Accrington).

All mosquitoes used for experiments, unless otherwise noted, were virgin and aged 3–6 days old. Female mosquitoes were used for all types of experiment conducted. Male mosquitoes were used for all types of experiment conducted, apart from experiments using step stimulation to investigate mechanical signatures of auditory transducer gating.

### Method details

#### Immunohistochemistry

Samples were prepared following previously published protocols.^9^^,^^50^ Heads were removed from adults from each genotype and fixed in 4% paraformaldehyde for an hour at room temperature; their proboscises were also removed prior to fixation. Following this, heads were embedded in albumin/gelatin and post-fixed overnight in 6% formaldehyde at 4 °C. Each block was then sectioned using a vibrotome into 40 μm sections and washed in phosphate-buffered saline 0.3% Triton X-100. Samples were blocked in 5% normal goat serum and 2% bovine serum albumin.

Primary antibodies used included: monoclonal antibody 3C11 (anti-SYNORF1; 1:50; Developmental Studies Hybridoma Bank, University of Iowa, http://dshb.biology.uiowa.edu/) and conjugated primary antibody anti-HRP-Cy3 (1:500, Jackson ImmunoResearch, Code: 123-165-021). Secondary antibodies used included corresponding Alexa Fluor Dyes (1:500; Thermo Fisher). Sections were mounted onto slides using DABCO and then visualised using a Zeiss 880 confocal microscope.

*Flagellar length measurements*: The right flagellae of adult mosquitoes from each genotype were removed using a pair of forceps whilst the mosquitoes were CO_2_ sedated. The flagellae were then transferred to separate microscope slides in groups of five. Each individual sample was then immediately imaged using a Zeiss Axioplan 2 microscope and Axiovision 4.3 software. Wing lengths were determined using Axiovision 4.3 software length measurement function, calibrated to the nearest 0.1 mm. Three biological repeats were conducted over different generations.

Total sample sizes for each group: *dsxF*^+/+^ *XX* = 59; *dsxF*^−/−^ *XX* = 40; *dsxF*^+/+^ *XY* = 56.

#### Laser Doppler Vibrometry - Mosquito preparation

As described previously, mosquitoes were glued to a Teflon rod using blue-light-cured glue.^9^ Glue was applied across the mosquito body to restrict movement, and thus disturbances, during recordings. The left flagellum was glued to the head before glue was applied between the pedicels. Only the right flagellum therefore was able to move completely unhindered.

The mounted mosquito was held in place by a micromanipulator placed on a vibration isolation table opposite a PSV-400 laser Doppler vibrometer (Polytec) with an OFV-70 close up unit and a DD-500 displacement decoder. The mosquito was orientated so as to face the laser Doppler vibrometer at a 90° angle. The laser was focused on the second flagellomere from the flagellum tip for all males, whilst the third flagellomere from the tip was used for all females. All LDV experiments took place in a temperature-controlled room (21°C–23°C).

#### Laser Doppler Vibrometry - Force step and sweeps procedure

A reference electrode was inserted into the thorax of a prepared mosquito to raise its’ electrostatic potential to −20 V relative to ground. A pair of electrostatic actuators were then positioned symmetrically around the unrestricted flagellum to enable electrostatic stimulation of the ear. A recording electrode was inserted at the base of the right pedicel to allow for recording of compound antennal nerve responses to the electrostatic stimulation.

The actuators were then used to provide stimulation to the flagellum. Flagellar displacement was monitored using the LDV, with simultaneous electrophysiological activity also recorded. Free fluctuations of the flagellum were recorded at the beginning and end of all experiments to assess the status of the mosquitoes’ auditory system.

Force step stimuli provided were identical to those reported previously^9^ and increased in intensity in logarithmic steps; 20 different intensities were used for each experiment (i.e., 20 different step magnitudes). Sweeps consisted of a pure tone stimulus which either increased linearly from 1 to 1000Hz over the course of 1 s (denoted as ‘forward sweep’), or decreased linearly from 1000 to 1Hz over the same time frame (denoted as ‘backward sweep’). Forward and backward sweeps were played sequentially, with a 1 s, unstimulated, pause in between each sweep. 10 different sweep intensities were used for each experiment, with data from the smallest and largest intensities used for analyses in Figure 2.

#### Laser Doppler Vibrometry - CO_2_ sedation experiments

Mounted mosquitoes were placed inside a rectangular steel chamber (6 × 6 × 2.5 cm^3^) positioned opposite the laser Doppler vibrometer. The chamber was held in place by a micromanipulator. The chamber side facing the vibrometer contained a glass window, thus enabling the recording of flagellar vibrations from mosquitoes.

Free fluctuation recordings were taken prior to CO_2_ exposure to test the baseline hearing status. CO_2_ was then allowed to flow into the chamber for one minute through the porous membrane floor. CO_2_ was maintained at a constant flow rate of 3 L/min throughout via a flow regulator (Flowbuddy, Flystuff). Looped free fluctuation recordings were taken whilst CO_2_ was allowed to flow into the chamber to monitor changes to the mosquito’s active hearing system. Once the active system was no longer visible (judged via reference to previous reports),^9^ CO_2_ flow was immediately stopped and a free fluctuation recording was taken. Each mosquito was given 5 minutes to recover from sedation, after which a final free fluctuation recording was taken.

Mosquito recovery from sedation was judged based on the final free fluctuation recording. Mosquitoes were deemed to have recovered if the best frequency and velocity amplitudes extracted from free fluctuation fits (described below) were altered from the baseline state by less than 20%. Those which did not fit the recovery criteria were excluded from all analyses. These recovery criteria were adopted for all LDV experiments in terms of comparing baseline and final free fluctuations.

#### Laser Doppler Vibrometry - Free fluctuation analysis

As reported previously,^9^ a forced damped harmonic oscillator function was fitted to flagellar velocity values obtained by applying Fast Fourier transforms (FFTs) to flagellar velocity amplitudes. The FFTs covered frequencies between 1 Hz and 10 kHz, though the function was only fit to values between 101 and 1000Hz due to significant levels of noise below 100Hz.

This function enabled estimation of the flagellar best frequency, as well as other parameters of interest. Analyses were aggregated across individual mosquitoes within a group in order to calculate population estimates and facilitate comparisons across groups.

Sample sizes for each group in the active state were: *dsxF*^+/+^ *XX* = 20; *dsxF*^−/−^ *XX* = 25; *dsxF*^+/+^ *XY* (non-SSO/SSO) = 11/21.

Sample sizes for each group in the passive state were: *dsxF*^+/+^ *XX* = 10; *dsxF*^−/−^ *XX* = 15; *dsxF*^+/+^ *XY* (non-SSO/SSO) = 10/11.

#### Laser Doppler Vibrometry - Apparent mass estimation

Apparent flagellar mass values are crucial for electrostatic stimulation analyses but can vary significantly across different mosquito sexes and species. We therefore calculated apparent mass values for each group of mosquitoes tested; samples sizes for each group were equal to the total number of passive state mosquito recordings per group.

We fitted the damped harmonic oscillator model described above to velocity spectra obtained from sedated mosquitoes and then calculated the apparent antennal mass based on the formula:

$$m=\frac{k_{B}T}{\omega_{0}^{2}\left\langle x^{2}\right\rangle }$$

where *m* represents the apparent antennal mass, *k*_*B*_ is the Boltzmann constant (1.38 × 10^−23^ J/K), T the absolute temperature (estimated at approximately 293 K), *ω*_*0*_ the natural frequency of the system and *<x*^*2*^*>* the flagellar receiver’s total fluctuation power. *ω*_*0*_ was calculated from the results of the damped harmonic oscillator function fit whilst $\left\langle x^{2}\right\rangle$ was calculated from:

$$\left\langle x_{i}^{2}\right\rangle =\int_{0}^{\infty }x_{i}^{2}\left(\omega \right)d\omega$$

#### Laser Doppler Vibrometry - Power gain

Power gain values were calculated by computing the ratio of total auditory system fluctuation power in active and passive states:

$$Powergain=\frac{E_{a}-E_{p}}{E_{p}}=\frac{\omega_{a}^{2}\left\langle x_{a}^{2}\right\rangle }{\omega_{p}^{2}\left\langle x_{p}^{2}\right\rangle }-1$$

where E_a_ and E_p_ represent the energy contents of the active and passive hearing systems, ω_a_ and ω_p_ the natural frequencies of the active and passive systems and $\left\langle x_{a}^{2}\right\rangle ,\left\langle x_{p}^{2}\right\rangle$ the total fluctuation powers of the active and passive systems respectively. Natural frequencies were calculated based on the results of the damped harmonic oscillator function fits in both active and passive states. Sample sizes for each group were equal to the number of passive state recordings taken per group.

#### Laser Doppler Vibrometry - Force step stimulation analysis

Force step analysis followed previously published protocols by utilising a two-state model of a single transducer population.^9^ Displacement data between ±2000 nm were analyzed for females in order to focus only on the most sensitive transducers.

Sample sizes for each group were: *dsxF*^+/+^ *XX* = 8; *dsxF*^−/−^ *XX* = 8.

#### Laser Doppler Vibrometry - Sweeps analysis

DC removes (with time constants of 0.015) were applied to flagellar displacement and nerve data in Spike2. Averages of flagellar displacement and nerve responses were then created in Spike2 for each stimulus type, with an average of each stimulus also created. These averages were then exported to Matlab for further analysis.

Maximal flagellar displacements were identified using a modified version of the ‘findpeaks’ function in MATLAB. As the change in stimulus frequency was linear, by identifying the timepoint at which this maximum was reached we were able to calculate the corresponding stimulus frequency. A similar procedure was used to identify the maximal nerve response and then estimate the stimulus frequency at which this occurred.

Mechanical sensitivities were calculated for each stimulus intensity by calculating the ratio of maximal flagellar displacement to stimulus magnitude (which remained constant throughout stimulus presentation). A three-parameter sigmoidal function was then fitted to the mechanical sensitivity values at each stimulus intensity, with all fits having calculated R^2^ values ≥ 0.9. Displacement gains were then calculated by computing the ratio of the maximal and minimal values obtained from the fit.

Sample sizes for each group: *dsxF*^+/+^ *XX* = 8; *dsxF*^−/−^ *XX* = 8; *dsxF*^+/+^ *XY* (SSO) = 10.

#### RNA sequencing - Sample collection

2-3 day old *dsxF*^+/+^ and *dsxF*^−/−^ *XX* and *XY* adult mosquitoes were placed into glass vials (as described above) in sex- and genotype-sorted groups of five. These vials were then transferred to the incubators described previously. Mosquitoes were entrained using a 12 h:12 h light/dark cycle for three days, with the first hour of light involving a constant increase in light intensity to a maximum and the last hour of light involving a constant decrease in light intensity to a minimum.

On the third day, vials containing mosquitoes were removed from the incubator during the period when light intensity began decreasing. Mosquitoes were immediately transferred to Eppendorfs and frozen in liquid nitrogen. Samples were stored in a −80°C freezer prior to dissections.

During dissections, both the mouthparts and flagellae were first removed from the head. Mosquito pedicels were then also removed from the head using a pair of forceps and transferred to an eppendorff containing 297 μL lysis buffer (InvitrogenTM PureLinkTM RNA Mini Kit) and 3 μL 2-beta-mercaptoethanol. Three biological repeats were collected for each group. Samples were then submitted for RNAseq total transcriptome Illumina sequencing at the Polo d'Innovazione di Genomica, Genetica e Biologia (PoloGGB).

The number of pedicels dissected for each repeat were: *dsxF*^+/+^ *XX* = 63/76/72 (repeat 1/2/3); *dsxF*^−/−^ *XX* = 70/72/72; *dsxF*^+/+^ *XY* = 71/64/64.

#### RNA sequencing - Data analysis

Raw read (fastq) files were first subjected to quality control using FastQC and MultiQC, and subsequently classified and quantified against the *An. gambiae* transcriptome (Anopheles-gambiae-PEST_TRANSCRIPTS_AgamP4.12.fa.gz obtained from VectorBase) using Kallisto. Furthermore, STAR was used in performing alignment of reads onto the An. gambiae genome (genome file: Anopheles-gambiae-PEST_CHROMOSOMES_AgamP4.fa.gz; annotation file: Anopheles-gambiae-PEST_BASEFEATURES_AgamP4.12.gtf.gz) to allow for visual inspection of the results using IGV. Quality control and differential expression analysis of read counts were conducted with the R-package DESeq2. The following comparisons were performed:

*dsxF*^+/+^ *XY* pedicels vs. *dsxF*^+/+^ X pedicels (M*v*F)
*dsxF*^−/−^ *XX* pedicels vs. *dsxF*^+/+^ *XX* pedicels (I*v*F)
*dsxF*^+/+^ *XY* pedicels vs. *dsxF*^−/−^ *XX* pedicels (M*v*I)

Transcripts were deemed to be significantly differentially expressed if FDR <0.05 (adjusted *p*-values from Wald tests). Where information was available, non-annotated transcripts were translated, aligned against the protein database UniProtKB reference proteomes plus Swiss-Prot of Uniprot, and assigned annotation based on homology. Scoring matrix used: BLOSUM-62. Homology thresholds: >33% query coverage, >33% identity between alignments, and E-value <0.0001.

GProfiler^51^ was used to conduct functional enrichment analysis on the genesets obtained by taking the intersections of the results of pairs of comparisons, namely the pairs: 1) MvF and IvF, and 2) MvF and MvI. The analysis was performed with emphasis on upregulated genes. Here upregulated refers to higher expression in the group on the left side of the comparison and characterized by a positive log2(FoldChange) (LFC). For example, if gene X has a positive LFC for the comparison MvF, that gene is upregulated in the male pedicel relative to the female pedicel. A gene ontology was deemed significantly enriched if the FDR <0.05 (g:SCS threshold). Testes comparisons were also conducted using GProfiler with the same analysis paradigm.

### Quantification and statistical analysis

Twenty step and free fluctuation analyses were conducted using Sigmaplot (Systat Software, Inc.). Remaining analyses were completed in MATLAB (Mathworks) and R. RNAseq analysis used R for differential gene expression, and GProfiler for functional enrichment.

Sample sizes for all experiments were determined via reference to published investigations.^9^^,^^50^ Within-group variation estimates were calculated when appropriate as part of standard statistical testing.

Statistical tests for normality (Shapiro–Wilk Normality tests with a significance level of *p* < 0.05) were first applied to each dataset. Throughout the analyses, all statistical tests used a significance level of *p* < 0.05.

Flagellar measurements were found to be not normally distributed; ANOVA on ranks tests followed by pairwise Mann-Whitney tests (with Bonferroni corrections when necessary) were thus used for comparisons across the genotypes and sexes.

Data obtained from the free fluctuation analyses were not found to be normally distributed. ANOVA on ranks tests followed by pairwise Mann-Whitney tests (with Bonferroni corrections when necessary) were therefore used for comparisons between genotypes and sexes, with comparisons for males split into SO and quiescent states. Paired Wilcoxon tests were used for comparisons within a mosquito group between active and passive states.

Data from the single transducer population fits were not found to be normally distributed. ANOVA on ranks tests followed by pairwise Mann-Whitney tests (with Bonferroni corrections when necessary) were therefore used to make comparisons between genotypes.

Best frequencies of both the mechanical and electrical responses to sweeps were not found to be normally distributed; Mann-Whitney tests (with Bonferroni corrections when necessary) were thus used for comparisons across genotypes and sexes. Paired Wilcoxon tests were used to compare between the largest and smallest intensity sweeps in terms of both mechanical and nerve best frequencies within a group.

## Supplemental information

Document S1. Figures S1–S6 and Table S1

Table S2. Raw RNA-seq count data for each genotype, related to Figures 3, S4, and S5

Table S3. Lists of genes for each subset investigated, as well as list of genes specifically highlighted in the manuscript, related to Figures 3 and S4 (A) List of genes included in *s*_*89*_. (B) List of genes included in *s*_*172*_. (C) List of genes included in *s*_*632*_. (D) List of genes included in *s*_*1092*_. (E) List of highlighted DEGs

Table S4. GO enrichment analysis outputs for each gene subset, related to Figures 3 and S5 (A) GO enrichment analysis for genes included in *s*_*89*_. (B) GO enrichment analysis for genes included in *s*_*172*_. (C) GO enrichment analysis for genes included in *s*_*632*_. (D) GO enrichment analysis for genes included in *s*_*1092.*_

Table S5. DESeq2 output for analysis of previously reported *Anopheles gambiae* Kisumu strain pedicel RNA-seq data, as well as the list of genes (with associated GO terms) overexpressed in both dsxF^+/+^ XY pedicels compared with dsxF^+/+^ XY and XY Kisumu pedicels compared with XX, related to Figure S6 (A) Deseq2 output for Kisumu XX and XY pedicels. (B) List of genes upregulated in both dsxF^+/+^ XY pedicels compared with dsxF^+/+^ XY and XY Kisumu pedicels compared with XX. (C) GO enrichment analysis of genes upregulated in both dsxF^+/+^ XY pedicels compared with dsxF^+/+^ XY and XY Kisumu pedicels compared with XX

Table S6. Lists of dyneins and tubulins expressed in male pedicels and testes, related to Figure 4 (A) List of tubulins. (B) List of dyneins
