# Supplementary material for: Using a female-specific isoform of doublesex to explore male-specific hearing in mosquitoes
Source: iScience. 2025 Aug 28;28(9):113330. doi: 10.1016/j.isci.2025.113330 (PMC12496202; doi:10.1016/j.isci.2025.113330)
Supplement: Document S1. Figures S1–S6 and Table S1 [file mmc1.pdf]

iScience, Volume *28*

Supplemental information

Using a female-specific isoform of *doublesex* to explore male-specific hearing in mosquitoes

Matthew P. Su, Marcos Georgiades, Marta Andrés, Jason Somers, Judit Bagi, YuMin M. Loh, Yifeng Y.J. Xu, Kyros Kyrou, Andrea Crisanti, and Joerg T. Albert

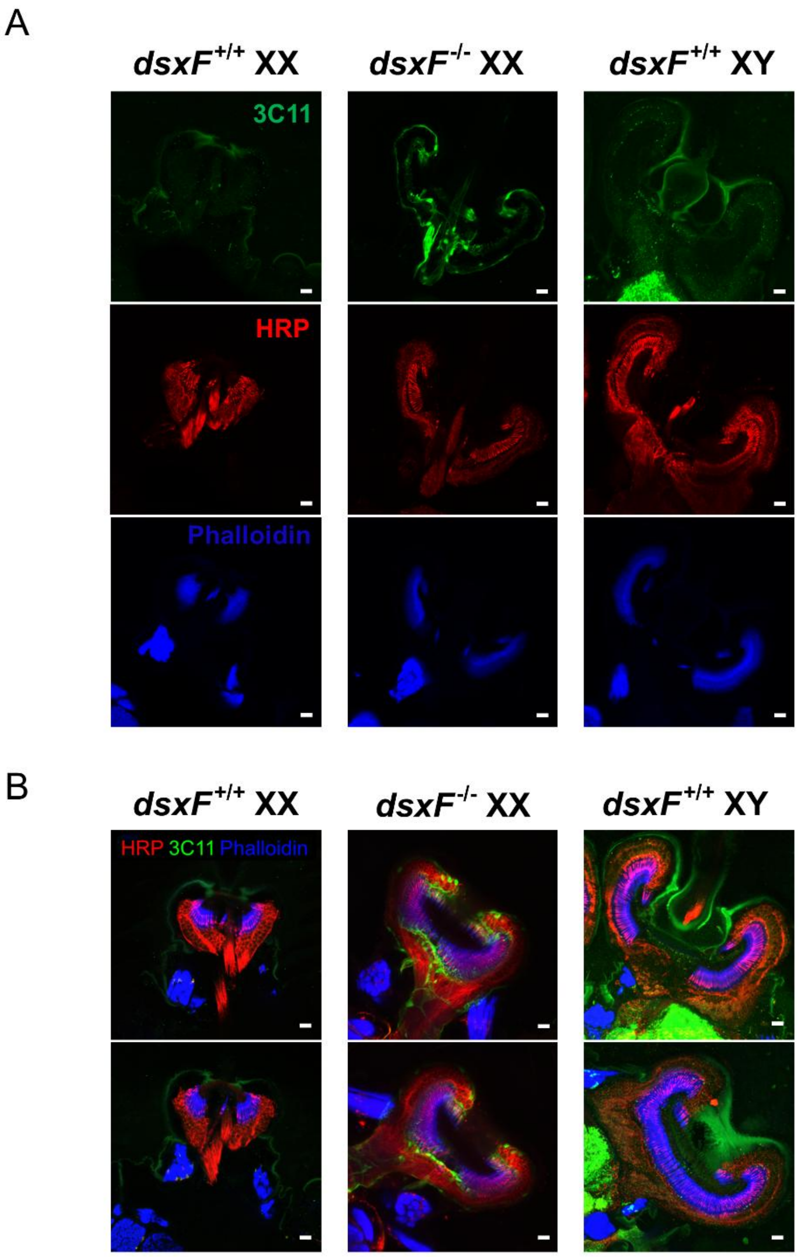

Supplementary Figure S1. ***Johnston's Organ sections for each genotype***

(A) Split channel images of horizontal sections of *dsxF*$^{+/+}$ XX (left), *dsxF*$^{-/-}$ XX (middle) and *dsxF*$^{+/+}$ XY (right) JOs from Fig 1B. Sections were stained with presynaptic marker 3C11 (anti-synapsin, green; top) to label presynaptic efferent terminals within the JO and counterstained with neuronal marker anti-HRP (red; middle) and phalloidin (blue; bottom) to label actin-based rods in scolopale and cap cells that surround auditory cilia. Scale bar: 10 µm.

(B) Further examples of sections of *dsxF*$^{+/+}$ XX (left), *dsxF*$^{-/-}$ XX (middle) and *dsxF*$^{+/+}$ XY (right) JOs. Sections were stained with presynaptic marker 3C11 (anti-synapsin, green) to label presynaptic efferent terminals within the JO and counterstained with neuronal marker anti-HRP (red) and phalloidin (blue) to label actin-based rods in scolopale and cap cells that surround auditory cilia. Scale bar: 10 µm.

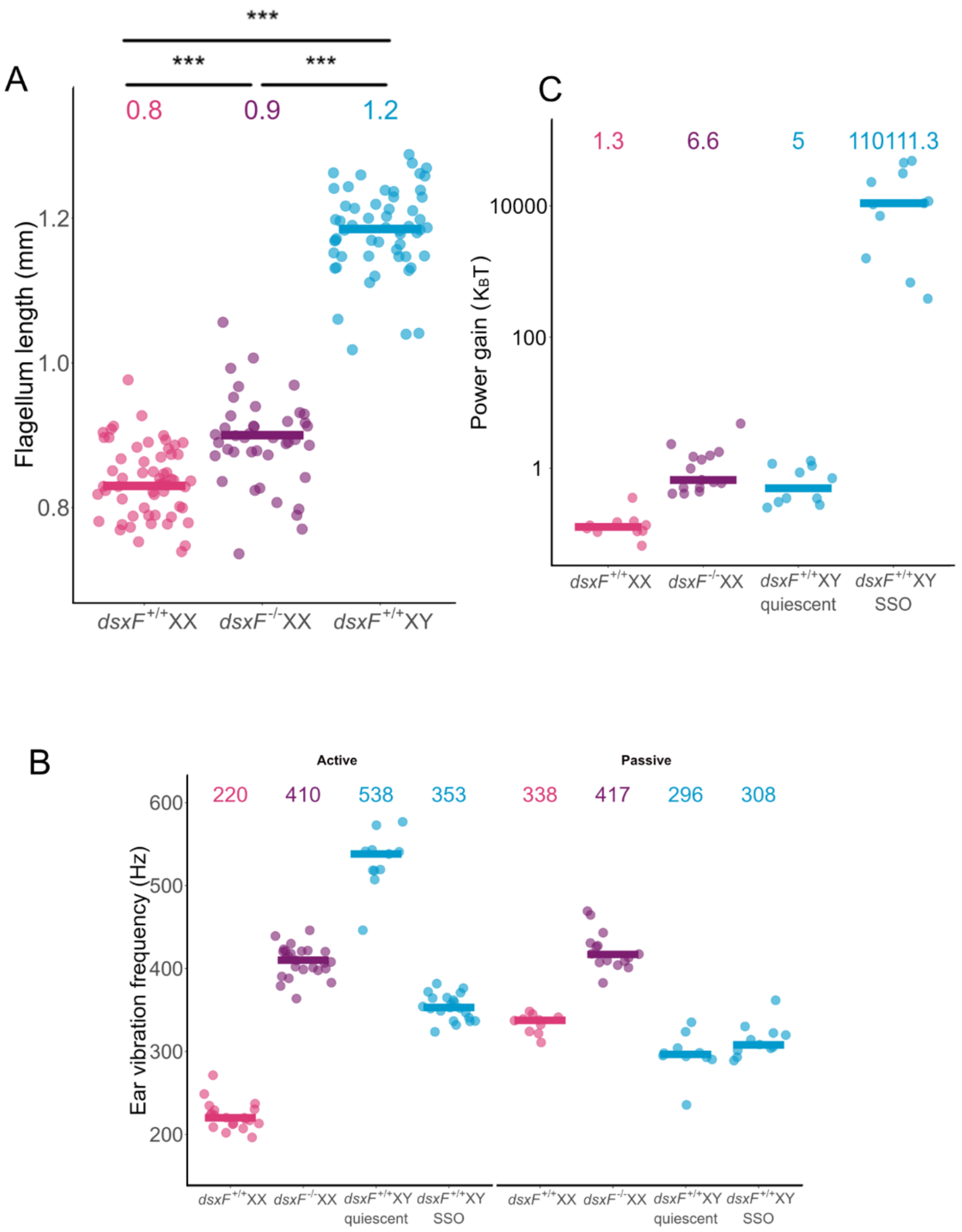

Supplementary Figure S2. ***Hearing function comparisons across groups including dsxF$^{+/+}$ XY showing SSOs***

(A) Flagellar length comparisons for each genotype. Individual points represent lengths of individual flagella. Median values are represented by solid lines, which are printed for each group at the top of the panel. Individual points are flagellar length measurements for individual mosquitoes from each group. Mann-Whitney tests with Bonferroni corrections used for statistical comparisons.

Sample sizes: *dsxF$^{+/+}$* XX = 59; *dsxF$^{-/-}$* XX = 40; *dsxF$^{+/+}$* XY = 56. ***, p<0.001.

(B) Calculated best frequencies of mosquito flagellar receivers for all groups including *dsxF$^{+/+}$* XY showing SSOs. Median values are represented by solid lines, which are

printed for each group at the top of the panel. Individual points are best mechanical tuning frequencies for individual mosquitoes from each group.

Sample sizes (females = active/ passive; males = active quiescent/ active SSO/ passive): *dsxF* $^{+/+}$ XX = 20/ 10; *dsxF*$^{-/-}$ XX = 25/ 15; *dsxF*$^{+/+}$ XY = 11/ 21/ 21.

(C) Calculated power gains for all groups including *dsxF*$^{+/+}$ XY showing SSOs. Median values are represented by solid lines, which are printed for each group at the top of the panel. Individual points are best mechanical tuning frequencies for individual mosquitoes from each group.

Sample sizes (females = active/ passive; males = active quiescent/ active SSO/ passive): *dsxF* $^{+/+}$ XX = 20/ 10; *dsxF*$^{-/-}$ XX = 25/ 15; *dsxF*$^{+/+}$ XY = 11/ 21/ 21.

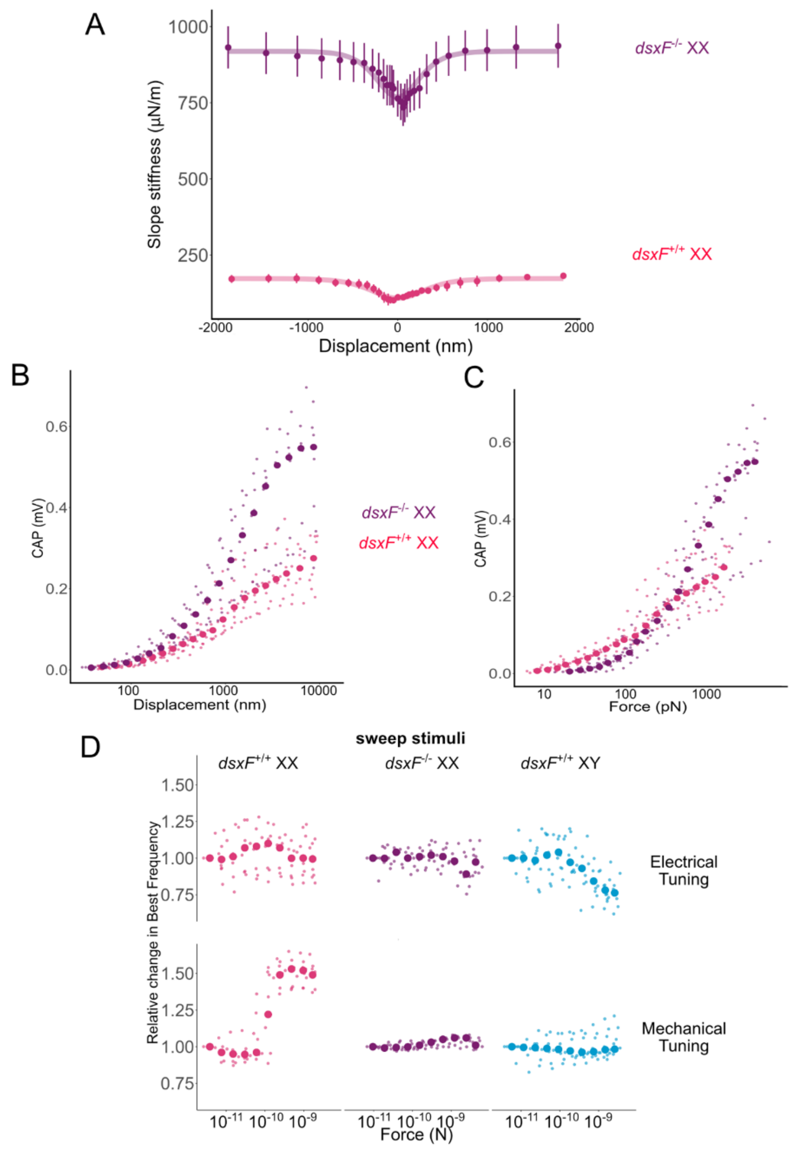

Supplementary Figure S3. ***Vibrometry/electrophysiology profiling of JO function***

(A) Median slope stiffness calculated for flagellar displacements in response to step stimulation for *dsxF*$^{+/+}$ (pink) and *dsxF*$^{-/-}$ XX (purple) mosquitoes. Solid lines represent the best fit of a single transducer population model for each genotype. Error bars represent ± SEM. Sample sizes: *dsxF*$^{+/+}$ XX = 8; *dsxF*$^{-/-}$ XX = 8.

(B,C) Median compound nerve responses in both (B) displacement and (C) force domains for *dsxF*$^{+/+}$ (pink) and *dsxF*$^{-/-}$ XX (purple) mosquitoes. Hollow, small points represent nerve responses for individuals whilst full, large points represent median responses per group. Sample sizes: *dsxF*$^{+/+}$ XX = 8; *dsxF*$^{-/-}$ XX = 8.

(D) Changes in (top) electrical and (bottom) mechanical best frequencies (BF) in response to increasing intensity of sweep stimulation for all genotypes. Hollow, small points represent relative changes for each individual relative to the smallest intensity whilst full, large points represent median changes per group. Left to right: *dsxF*$^{+/+}$ XX; *dsxF*$^{-/-}$ XX; *dsxF*$^{+/+}$ XY. Sample sizes: *dsxF*$^{+/+}$ XX = 8; *dsxF*$^{-/-}$ XX = 8; *dsxF*$^{+/+}$ XY SSO = 10.

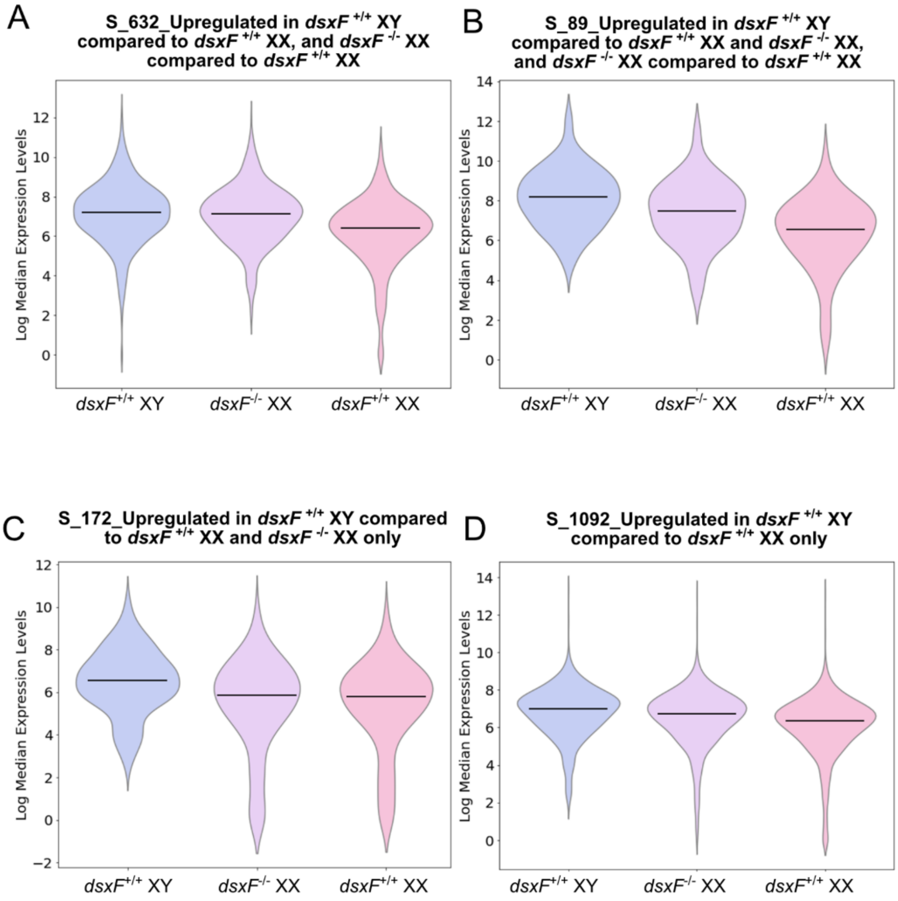

Supplementary Figure S4. ***Log median expression of genes in different comparison subsets***

(A) Log median expression levels of genes only upregulated in *dsxF*$^{+/+}$ XY pedicels compared to *dsxF*$^{+/+}$ XX and *dsxF*$^{-/-}$ XX pedicels (s_{632}).

(B) Log median expression levels of genes upregulated in *dsxF*$^{+/+}$ XY pedicels compared to *dsxF*$^{+/+}$ XX and *dsxF*$^{-/-}$ XX pedicels, and *dsxF*$^{-/-}$ XX pedicels compared to *dsxF*$^{+/+}$ XX pedicels (s_{89}).

(C) Log median expression levels of genes upregulated in *dsxF*$^{+/+}$ XY pedicels compared to *dsxF*$^{+/+}$ XX pedicels, and *dsxF*$^{-/-}$ XX pedicels compared to *dsxF*$^{+/+}$ XX pedicels (s_{172}).

(D) Log median expression levels of genes only upregulated in *dsxF*$^{+/+}$ XY pedicels compared to *dsxF*$^{+/+}$ XX pedicels (s_{1092}).

The panels here serve as a visual supplement to the functional and/or regulatory interpretations made for each subset of the 1985 transcripts upregulated in the M*v*F comparison. For example, it can be seen from panel (A) that transcript members of this set are upregulated in *dsxF*$^{+/+}$ XY compared to *dsxF*$^{+/+}$ XX and in *dsxF*$^{-/-}$ XX compared to *dsxF*$^{+/+}$ XX, but are, on average, not different between *dsxF*$^{+/+}$ XY and *dsxF*$^{-/-}$ XX.

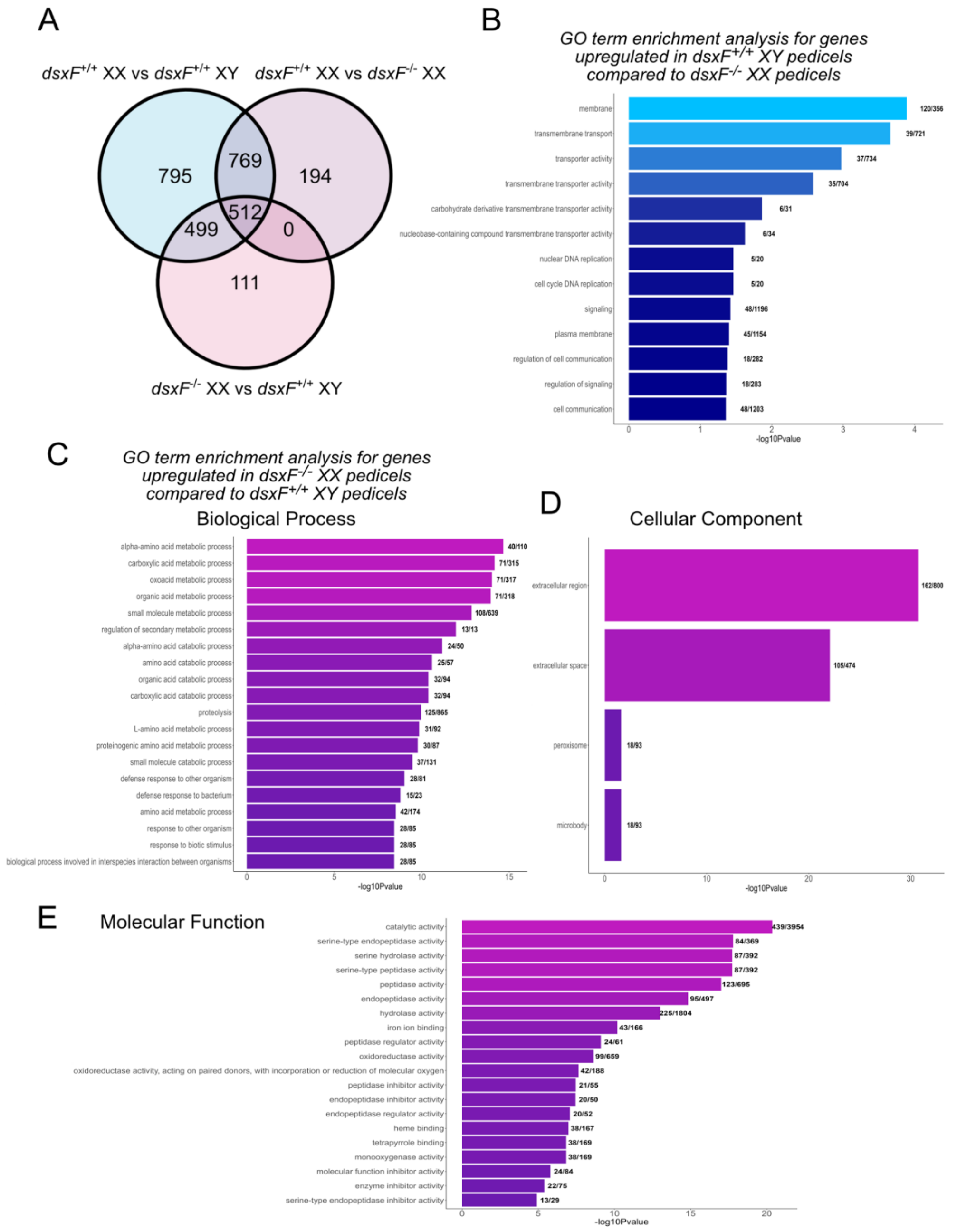

Supplementary Figure S5. *Supplemental gene ontological analysis for differentially expressed genes*

(A) Venn Diagram of gene intersections showing those differentially regulated between *dsxF*$^{+/+}$ XY, *dsxF*$^{-/-}$ XX and *dsxF*$^{+/+}$ XX pedicels. Upregulated group is to left hand side of group name.

(B) GO term enrichment analysis (Biological Process, Cellular Component and Molecular Function) for genes upregulated in *dsxF*$^{+/+}$ XY pedicels compared to *dsxF*$^{-/-}$ XX.

(C-E) GO term enrichment analysis for genes upregulated in *dsxF*$^{-/-}$ XX pedicels compared to *dsxF*$^{+/+}$ XY. (C), Biological Process; (D), Cellular Component; (E), Molecular Function.

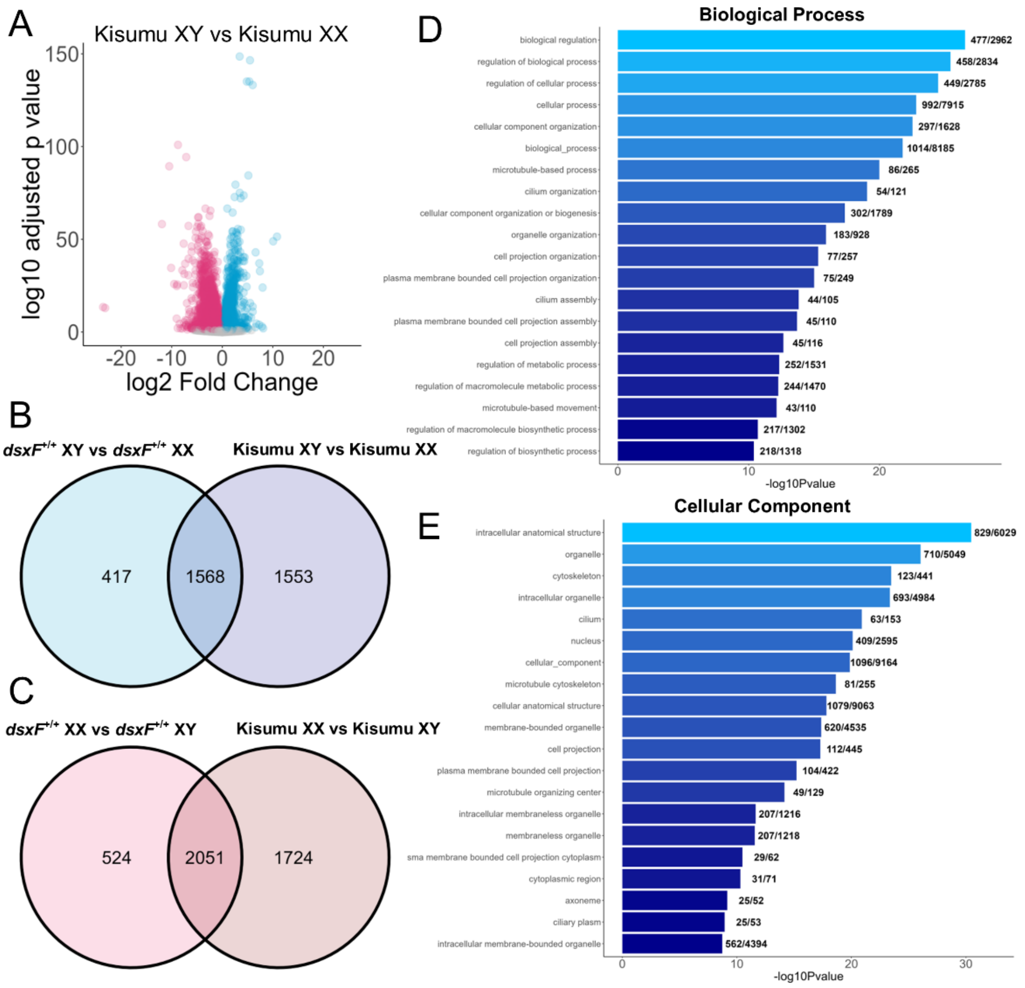

Supplementary Figure S6. ***Analysis of previously reported Anopheles gambiae Kisumu strain pedicel RNAseq data***

(A) Volcano plot of differential gene expression between *Anopheles gambiae* Kisumu strain XY and XX pedicels.

(B) Venn Diagram of genes upregulated in either *dsxF*$^{+/+}$ XY pedicels compared to *dsxF*$^{+/+}$ XX, Kisumu strain XY compared to XX pedicels, or both sets. Upregulated group is to left hand side of group name.

(C) Venn Diagram of genes upregulated in either *dsxF*$^{+/+}$ XX pedicels compared to *dsxF*$^{+/+}$ XY, Kisumu strain XX compared to XY pedicels, or both sets. Upregulated group is to left hand side of group name.

(D) GO term enrichment analysis (Biological Process) for genes upregulated in both *dsxF*$^{+/+}$ XY pedicels compared to *dsxF*$^{+/+}$ XX and Kisumu strain XY pedicels vs XX.

(E) GO term enrichment analysis (Cellular Component) for genes upregulated in both *dsxF*$^{+/+}$ XY pedicels compared to *dsxF*$^{+/+}$ XX and Kisumu strain XY pedicels vs XX.

Supplementary Table S1: Parameter values for single transducer population model fits to median slope stiffness values

N is the estimated transducer channel number and z is the change in force after one gating spring opens a transducer channel. $K_{INFINITY}$ represents the asymptotic flagellar stiffness for large displacements. K_{STEADY} represents the combined elasticity of the flagellar joint and receiver neurons. K_{GS} (also referred to as the gating spring stiffness) was calculated as $K_{INFINITY} - K_{STEADY}$ and represents the mechanical integrity of the transducer modules themselves. The extent of nonlinearity indicates how nonlinear the system is. CAP_{50} values represent the minimum flagellar displacement required to produce 50% of the maximum nerve response. Significant differences found between *dsxF*$^{+/+}$ *XX* mosquitoes and *dsxF*$^{-/-}$ *XX* are starred (Mann-Whitney tests; *$p < 0.05$).

	dsxF $^{+/+}$ *XX*	*dsxF* $^{-/-}$ *XX*
Sample size	8	8
Channel number, N	3130.93 (594.94)	1704.33 (632.16)
Channel gating force, z (fN)	24.21* (1.31)	33.03 (4.59)
$K_{INFINITY}$ (μN/m)	308.65* (15.27)	918.43 (67.76)
K_{STEADY} (μN/m)	192.11* (7.74)	633.82 (57.77)
K_{GS} (μN/m)	98.76* (10.52)	283.26 (30.46)
Extent of nonlinearity	0.411* (0.06)	0.153 (0.02)
CAP_{50} (nm)	992.50 (102.25)	1065.42 (88.09)
